# Selenium and selenoproteins in viral infection with potential relevance to COVID-19

**DOI:** 10.1016/j.redox.2020.101715

**Published:** 2020-09-10

**Authors:** Jinsong Zhang, Ramy Saad, Ethan Will Taylor, Margaret P. Rayman

**Affiliations:** a Key Laboratory of Tea Plant Biology and Utilization, School of Tea & Food Science, Anhui Agricultural University, 130 West Changjiang Road, Hefei, 230036, Anhui, PR China; b Department of Nutritional Sciences, Faculty of Health and Medical Sciences, University of Surrey, Guildford, GU2 7XH, UK; c Royal Sussex County Hospital, Brighton, BN2 5BE, UK; d Department of Chemistry and Biochemistry, University of North Carolina Greensboro, Greensboro, NC 27402, USA

**Keywords:** SARS-CoV-2, COVID-19, Selenium, Selenoproteins, Redox-active selenium species, Ebselen

## Abstract

Selenium is a trace element essential to human health largely because of its incorporation into selenoproteins that have a wide range of protective functions. Selenium has an ongoing history of reducing the incidence and severity of various viral infections; for example, a German study found selenium status to be significantly higher in serum samples from surviving than non-surviving COVID-19 patients. Furthermore, a significant, positive, linear association was found between the cure rate of Chinese patients with COVID-19 and regional selenium status. Moreover, the cure rate continued to rise beyond the selenium intake required to optimise selenoproteins, suggesting that selenoproteins are probably not the whole story. Nonetheless, the significantly reduced expression of a number of selenoproteins, including those involved in controlling ER stress, along with increased expression of IL-6 in SARS-CoV-2 infected cells in culture suggests a potential link between reduced selenoprotein expression and COVID-19-associated inflammation. In this comprehensive review, we describe the history of selenium in viral infections and then go on to assess the potential benefits of adequate and even supra-nutritional selenium status. We discuss the indispensable function of the selenoproteins in coordinating a successful immune response and follow by reviewing cytokine excess, a key mediator of morbidity and mortality in COVID-19, and its relationship to selenium status. We comment on the fact that the synthetic redox-active selenium compound, ebselen, has been found experimentally to be a strong inhibitor of the main SARS-CoV-2 protease that enables viral maturation within the host. That finding suggests that redox-active selenium species formed at high selenium intake might hypothetically inhibit SARS-CoV-2 proteases. We consider the tactics that SARS-CoV-2 could employ to evade an adequate host response by interfering with the human selenoprotein system. Recognition of the myriad mechanisms by which selenium might potentially benefit COVID-19 patients provides a rationale for randomised, controlled trials of selenium supplementation in SARS-CoV-2 infection.

## Highlights

• Many RNA (or reverse transcribing) viruses are more pathogenic in Se deficient hosts.
• Both as dietary factor and pharmacological agent, Se can protect against some viruses.
• Various selenoproteins contribute to host immunity & play roles in viral pathogenesis.
• Se adequacy can prevent excessive cytokine activation and enhance adaptive immunity.
• Redox-active Se metabolites may explain some benefits of Se at supranutritional doses.

## 1 Introduction

Selenium (Se) is a unique trace element; it is the only one of the trace elements to be specified in the genetic code. It is essential at a very low level of intake, from 55 to 75 μg/d [1,2], yet toxic above 800 μg/d, with the Safe Upper Limit being defined as 400 μg/d [1]. The essentiality of selenium is linked to the remarkable range of functions of the selenoproteins that are described below [3].

Selenium gets into the food chain through plants which take it up from the soil. The amount taken up is dependent not only on the selenium content of the soil which relates to the underlying geology, but to soil pH, the presence of organic matter and climatic conditions [4]. The effect of climate is nicely exemplified by the selenium-poor belt in China where the selenium status is decisively affected by monsoonal precipitation [5].

An unusual aspect of selenium is the extremely wide range of intake seen across the globe (Supplemental Table 1 [6,7]); that in China varies from the lowest to the highest in the world. Intake currently ranges from 14 μg/d in Mianning County, Sichuan Province which has a long history of selenium deficiency and was the site in 1974–1976 of a study showing that selenium supplementation prevents Keshan disease [8], to 550 μg/d in Enshi County, Hubei Province (Supplemental Table 1 [6,7]). In the 1960s, Se toxicity (selenosis) was prevalent in Enshi County and, as late as 1981, intake was reported to be as high as 4990 μg/d in some areas of Enshi [9].

With this remarkable degree of variation in intake, it is not surprising that there have been numerous examples of adverse health conditions linked to selenium deficiency including those caused by viruses [3,10] and also by selenium excess [11]. As with many nutrients, there is a U-shaped relationship between Se intake or status and its health effects; that relationship is particularly noticeable for selenium [11].

In this comprehensive review we will explore the evidence for the involvement of selenium, whether as particular selenium species or selenoproteins, in viral infections. We will first cover the important role of the selenoproteins in combatting viral infection. We will then describe the evidence for the effects of selenium/selenoproteins on viral pathogenicity, including data that link them with SARS-CoV-2/COVID-19. We will discuss the effect of selenium/selenoproteins on immunity and production of inflammatory cytokines and will touch on the possible viral targeting of selenoprotein mRNAs by antisense and related mechanisms. We will then consider potential mechanisms by which selenium species, including selenoproteins, might affect COVID-19 outcome, mentioning specifically the synthetic selenium compound, ebselen, and redox-active selenium species. We will finish by discussing whether selenium supplementation might potentially benefit SARS-CoV-2 infected individuals, and if so, what the relevant dose might be.

## 2 The selenoproteins

All the selenoproteins contain selenocysteine at their active centre and are synthesised by a complex process [12]. In the presence of a selenocysteine insertion sequence (SECIS) in the 3′-untranslated region of mRNA, the UGA codon, which normally acts as a stop codon, is recoded to specify the insertion of selenocysteine [12]. A number of other factors are also required before selenocysteine can be incorporated into the growing protein chain [12]. The human genome contains 25 genes that encode selenoproteins. These selenoproteins have a wide range of functions, from antioxidant and anti-inflammatory roles to the production of active thyroid hormone [12,13]. Many selenoprotein genes are polymorphic; where the genotype of a particular polymorphism (or SNP) affects the risk of a health condition, we know that the function of that selenoprotein is relevant to that condition [3].

### 2.1 Selenoprotein functions relevant to viral infection

Selenoprotein functions known to be relevant to viral infection are shown in Table 1. From the table, it is clear that there are many ways in which selenium, *via* selenoprotein functions, might counteract infection with SARS-CoV-2.

**Table 1:** Multiple selenoprotein functions relevant to viral infection.

Selenoprotein function	Selenoproteins that carry out those functions, with some examples
Antioxidant	The following selenoproteins have antioxidant functions: **GPX1, GPX2, GPX3, GPX4, TXNRD1, TXNRD2, TXNRD3, MSRB1, SELENOP, SELENOW** [3,14,15]
	**GPX1** protects against Keshan Disease [16].
	***GPx1*** Pro198Leu SNP affects risk of Kashin-Beck disease [17].
	***GPX4*** rs713041 SNP affects risk of pre-eclampsia [18].
	**GPX4** reduces lipid peroxides accumulated during ferroptosis into non-toxic lipid alcohols [19].
Redox function; maintaining cellular redox homeostasis	**TXNRD1** required for DNA synthesis [20].
	**TXNRD1** maintains redox tone in immune cells through regeneration of reduced cytosolic TXN1 [15].
	**TXNRD2** preserves mitochondrial integrity, redox homeostasis and cardiac function in the ageing heart [21].
Anti-inflammatory	**Selenoproteins:**
	–produce anti-inflammatory lipid mediators from arachidonic acid to protect cells against pro-inflammatory gene expression induced by oxidative stress [14,22]
	–increase the production of 15d-PGJ2 decreasing activation of NF-κB and down- regulating inflammatory-gene expression [14,22]
	–activate PPAR-γ, repressing inflammatory gene expression [14,22]
	**GPXs:** metabolise ROS to prevent activation of NF-κB, its translocation to the nucleus and its binding to pro-inflammatory cytokine genes [23].
	**TXNRD1:** induces haem oxidase-1 which has anti-inflammatory functions linked to its removal of the pro-oxidant, haem, its production of the antioxidant biliverdin and the vasodilatory, anti-inflammatory carbon monoxide [22,24].
	**SELENOS**: reduces inflammation by removing misfolded proteins from the ER, protecting from the unfolded protein response [25]. See data on *SELENOS* SNPs –rs28665122 (autoimmune thyroid disease, pre-eclampsia) [26,27]; rs8025174 (CHD); rs7178239 (ischemic stroke) [28].
Immune-cell function	**Selenoproteins:**
	– regulate inflammation and immunity, being linked to redox signalling, oxidative burst, calcium flux, and the subsequent effector functions of immune cells [15]
	– induce up-regulation of the IL-2 receptor increasing the ability of T and B lymphocytes to respond to IL-2, and augmenting immune-cell function [29].
	**GPXs, TXNRDs and MSRB1** maintain redox tone or reverse oxidative damage inflicted on immune cells, for instance, from their own respiratory-burst reaction [15].
	**SELENOK** is required for the ER associated protein degradation (ERAD) pathway and regulation of Ca^2+^ flux from the ER. Several immune cell functions rely on efficient store operated Ca^2+^ entry (SOCE) and are compromised in SELENOK deficient immune cells; these include proliferation, migration, cytokine secretion and protection against pathogens [15,30].
	**SELENOS** mitigates ER stress arising from increased protein processing that accompanies macrophage activation [15,30].
	**SELENOS** affects circulating levels of inflammatory cytokines (IL-1β, IL-6, TNF-α) involved in Hashimoto's Thyroiditis pathogenesis [25,26].
	**GPX3** removes excessive H_2_O_2_, protecting against autoimmune thyroid disease (Hashimoto's Thyroiditis, HT) [31].
	**GPX4** plays an essential role in T cell immunity by preventing ferroptotic cell death [32].
Antiviral effects	Se/**selenoproteins** such as **GPXs, TXNRDs** and **ER selenoproteins** influence viral pathogenicity, partly by reducing oxidative stress generated by viral pathogens as shown in the following viral infections: coxsackievirus B3, Influenza A/Bangkok/1/79 (H3N2), Influenza H1N1, HIV-1, Polio, Hepatitis B & C, Hantavirus [33]. Inability to counteract oxidative stress can result in mutations in the viral genome from benign to highly virulent [16,33].
	**TXNRD1** is critical for expansion of the activated T-cell population during infection with lymphocytic choriomeningitis virus (LCMV)-WE strain [20].
	***GPX1*** (Pro198Leu) polymorphism is implicated in the severity of liver fibrosis and hepatocellular carcinoma caused by hepatitis C virus [34].
	**Selenoproteins** can be encoded in the viral genome, e.g. in Molluscum Contagiosum & Fowlpox, presumably protecting them against ROS produced by host phagocytes [33].
	NF-κB is activated by multiple families of viruses, promoting viral replication and preventing virus-induced apoptosis [35]. By increasing the production of 15d-PGJ2, Se/**selenoproteins** can decrease activation of NF-κB, reducing viral replication [14].
Transport	**SELENOP** transports Se from liver to tissues including brain, testes and placenta [36].
Protection of the cardiovascular system	**GPX1**: protects against cardiomyopathy in Keshan Disease [16]; higher GPX1 activity significantly reduced the risk of a cardiovascular event in patients with coronary artery disease [37].
	***GPX1*** 198Pro/Leu variant genotype significantly associated with coronary artery disease risk in a Chinese population [38].
	***GPX4***: individuals with the C718T TT genotype have impaired endothelial function and greater risk of vascular disease [39].
	**TXNRD2**: preserves mitochondrial integrity, redox homeostasis, and cardiac function in the ageing heart [21].

Se = selenium; GPX1, GPX2, GPX3, GPX4 = cytosolic, gastrointestinal, extracellular, phospholipid glutathione peroxidases, respectively; TXNRD1, TXNRD2, TXNRD3 = cytosolic, mitochondrial, testis thioredoxin reductases, respectively; MSRB1 = methionine sulfoxide reductase B1; SELENOP = selenoprotein P; SELENOK = selenoprotein K; SELENOW = selenoprotein W; PPAR-γ = Peroxisome proliferator-activated nuclear receptor-γ; IKKβ = IκB-kinase β.

### 2.2 Effect of SARS-CoV-2 on selenoprotein expression

There is already some evidence for a link between SARS-CoV-2 infection and selenoproteins; infection of cultured Vero E6 cells with SARS-CoV-2 significantly reduced the expression of a number of selenoproteins (GPX4, TXNRD3, and the endoplasmic reticulum selenoproteins, SELENOS, SELENOK, SELENOF, SELENOM) while increasing the expression of the inflammatory cytokine, IL-6 [40]. Concomitant down-regulation of *SELENOF*, *SELENOM*, *SELENOK* and *SELENOS* by SARS-CoV-2 is likely to result in increased concentration of misfolded proteins in the ER and catastrophic ER stress. A direct mechanistic link between the reduced expression of *SELENOS* and the production of inflammatory cytokines has already been well documented [25]; such a mechanism may be relevant to the marked elevation of IL-6 concentration induced by SARS-CoV-2.

## 3 Selenium status and viral pathogenicity

Significant clinical benefits of selenium supplementation have been demonstrated in a number of viral infections, as previously reviewed [33,41,42]. These include most notably, coxsackievirus B3 and Keshan Disease, a cardiomyopathy named after the area in north-east China where it was endemic [16]; when the population was supplemented with selenium enabling adequate synthesis of the antioxidant GPX1 to occur, the incidence of Keshan Disease fell from 11.2 cases/1000 to 0.6 cases/1000 [3,43]. In a series of clever experiments, Beck and co-workers showed that in mice that were unable to make sufficient GPX1, both coxsackievirus B3 and influenza A/Bangkok/1/79 (H3N2) mutated into more virulent forms that caused more severe disease or death [16]. Some other examples of the effects of selenium are in HIV-1, where a negative correlation between selenium status and mortality has been established [3,44], in hepatitis B linked to liver cancer [45], and in patients with hantavirus (“epidemic hemorrhagic fever”) who were successfully treated with oral sodium selenite, giving an overall 80% reduction in mortality [46]. From the above data it seems clear that selenium intake or status is relevant to infection with a number of evolutionarily distinct viruses and that selenium deficiency can influence viral mutation and evolution [16].

Factors associated with COVID-19-related death include male sex, age, deprivation, ethnicity, obesity, diabetes, severe asthma and other chronic diseases [47]. With regard to sex, there appear to be key differences in the baseline immune capabilities in men and women during the early phase of SARS-COV-2 infection, with female patients mounting significantly more robust T-cell activation than male patients [48]. However, it is the case that a number of these factors, most notably age, obesity and chronic disease, for instance, chronic obstructive pulmonary disorder (COPD), also have a negative effect on selenium status [47,[49], [50], [51]], suggesting that such patients might benefit from nutritional levels of selenium supplementation. However, the low selenium status linked to these conditions may well be a by-product of the associated inflammation [[52], [53], [54]] and factors other than selenium are likely to be much more relevant.

**Fig. 1:**
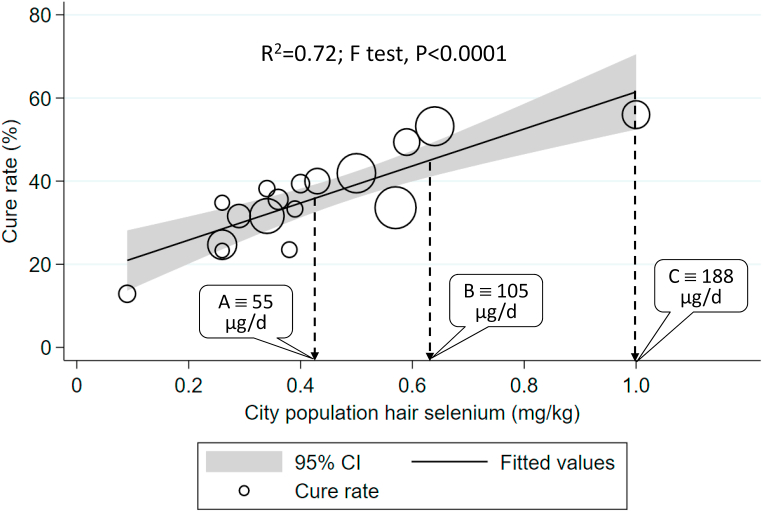
Correlation between COVID-19 cure rate in 17 cities outside Hubei on Feb 18, 2020, and city population selenium status (hair selenium concentration) analysed using weighted linear regression. Each data point represents the cure rate, calculated as percentage of patients hospitalized with SARS-CoV-2 deemed to be cured*. The size of the marker is proportional to the number of cases (adapted from Am J Clin Nutr [7] with permission). From the graph of Se intake vs hair Se concentration, Supplemental Figure 1, Se_intake_ = 232.98 Se_hair_ – 44.521,allowing the calculation of corresponding values of selenium intake and hair concentration (Supplemental Table 3). Thus value A represents the hair concentration corresponding to an intake of 55 µg/d where platelet GPX1 activity is maximised [58], value B represents the hair concentration corresponding to an intake of 105 µg/d where SELENOP concentration is maximised [58], and value C is the hair selenium concentration (1.0 mg/kg) at the maximum cure rate in the investigated cities which corresponds to an intake of 188 µg/d.*Cured patients are those in whom temperature has returned to normal for more than 3 days, respiratory symptoms are significantly improved, lung imaging shows significant reduction of inflammation, negative nucleic acid test of respiratory pathogen on two consecutive occasions with a sampling interval of at least 1 day.

The most recent example of the relevance of selenium to viral diseases is our analysis of the COVID-19 cumulative data on the specific date of February 18, 2020 in Chinese cities [7]. On inspection of the data from Hubei province where the first cases were reported, it was notable that the cure rate in Enshi city, renowned for its high selenium intake (550 μg/d in 2013 [55]), at 36.4%, was much higher than that of other Hubei cities where the overall cure rate was 13.1%; indeed, the Enshi cure rate was significantly different from that in the rest of Hubei (p < 0.0001) [7]. Similar inspection of data from provinces outside Hubei showed that Heilongjiang Province in north-east China, a notoriously low-selenium region (intake 16 μg/d in 2018 [56]), had a much higher death rate, at 2.4%, than that of other provinces (0.5%); p < 0.0001 [7]. When we plotted the cure rate in cities outside Hubei province against population selenium status, as measured by hair selenium concentration (a validated measure of selenium intake [57]), we found a significant linear association; R^2^ = 0.72, F-test p < 0.0001 [7] (Fig. 1).

We were interested to see what range of selenium intake was represented by the hair-selenium concentrations in Fig. 1, so we searched the literature for data on selenium intake that had corresponding values of hair-selenium concentration (see Supplemental Table 2). The regression of selenium intake against hair selenium (Supplemental Fig. 1) enabled us to determine the hair-selenium concentration corresponding to an intake of 55 μg/d, at which platelet GPX1 activity is maximised [58], i.e. 0.43 mg/kg (see Supplemental Table 3). We similarly determined the hair selenium concentration corresponding to an intake of 105 μg/d at which SELENOP concentration is optimised, i.e. 64 mg/kg [58]. Interestingly, there is no sign of a plateau in Fig. 1 at either of those hair-selenium concentration values. Indeed, the cure rate continues to rise above those points, reaching the high end of the regression line at a cure rate of 56% and hair-selenium concentration of 1.0 mg/kg, corresponding to an intake of 188 μg/d (see Supplemental Table 3) suggesting that selenoproteins are unlikely to be the whole story.

Some readers may question why, if COVID-19 outcome is related to selenium status, we have not seen better disease outcomes in countries with higher selenium status, such as the USA, Canada and Japan, than in countries of Europe that generally have lower status. While acknowledging differences in a number of factors that vary between countries including rate of testing, demographics and healthcare-system characteristics, as already pointed out by Seale and colleagues [59], countries with the highest reported COVID-19 case-fatality rates [60] correspond to those where suboptimal selenium status has previously been documented [6,61]. Thus, mean (SD) case-fatality rates in Italy, France, Spain and the United Kingdom were recorded by the Johns Hopkins Coronavirus Resource Centre as 13.4 (2.1)% [corresponding to a mean (SD) intake of 56 (8) μg/d] on July 28, 2020 whereas the corresponding value in the United States, Canada and Japan where the populations are selenium adequate, was substantially lower, at 4.8 (2.5)% [corresponding to a mean (SD) intake of 128 (46) μg/d] (see Supplemental Table 1) [6,60]. As to why we saw an apparently unique association between selenium status and COVID-19 cure-rate in Chinese cities, it may relate to the fact that the maximum and minimum selenium intakes in China are more widely separated, at 14 and 550 μg/d, than intakes elsewhere in the world [7].

Reinforcing our observed association between selenium status and outcome of COVID-19 disease is a German study that found a pronounced deficit in total serum selenium and SELENOP concentrations in COVID-19 patients when compared with reference data from the large European EPIC cross-sectional study [62]. Selenium status below the 2.5th percentile of the reference population was present in 43.4% (selenium) and 39.2% (SELENOP) of COVID patient serum samples. Furthermore, selenium status was significantly higher in samples from surviving COVID patients than from non-survivors (selenium, 53.3 ± 16.2 *vs*. 40.8 ± 8.1 μg/L; SELENOP, 3.3 ± 1.3 *vs*. 2.1 ± 0.9 mg/L) [62]. As this is an observational study, it cannot show causality; the low selenium status associated with severity could reflect reduced expression of *SELENOP* by inflammatory cytokines under acute-phase conditions [62]. However, the exceptionally low level of serum selenium reached will inevitably have adverse effects on the concentration of protective selenoproteins which, according to Moghaddam and colleagues [62], argues for the potential relevance of some supplemental selenium support in severe COVID disease. One might speculate that such low selenium status in severe COVID-19 disease could potentially be linked *via* reduced selenoprotein deiodinase activity to the low thyroid function (serum total tri-iodothyronine) observed in COVID patients that correlated significantly with disease severity [63].

## 4 Potential mechanisms by which selenium species could affect SARS-CoV-2 or COVID-19

We have presented above the evidence that shows an association between selenium species and SARS-CoV-2 or COVID-19 disease. While there is as yet no evidence for causality, there are a number of plausible mechanisms by which selenium, in one or other of its forms, could affect the virus and indeed vice-versa. These will be explored below beginning with immune system effects.

### 4.1 The immune system in COVID-19

Our understanding of the full role the immune system plays in COVID-19 is still developing; however, there is mounting evidence that excessive innate responses and cytokine release contribute to morbidity and mortality. It has been noted that severe disease correlates with tissue accumulation of macrophages and the production of the inflammatory cytokines IL-1β, IL-6, and TNF-α [64]. “Functional exhaustion” of natural killer (NK) Cells and cytotoxic T-cells has also been identified as a factor in severe COVID-19 [65].

Several features of severe COVID-19 display signs of immunological dysfunction – namely lymphopenia, high levels of inflammatory cytokines and monocytic invasion of pulmonary tissue [66]. Curiously, simultaneous occurrences of immunological diseases in patients with COVID-19 are surfacing, indicating disarray in both adaptive and humoral immunity. These include cases of the Anti-Phospholipid Syndrome (APLS) [67], five documented cases of Guillain-Barré Syndrome (GBS) [68] and a case of Immune Thrombocytopenia Purpura (ITP), that appeared to be directly triggered by the SARS-COV-2 virus [69]. Furthermore, some of the rashes apparent in COVID-19 patients have a resemblance to those associated with immune-complex deposition, such as chilblains and livedo reticularis [70].

Selenium supplementation has been noted to stimulate T-cell proliferation and enhance innate immune-system functions [71]. Supplementation of 200 μg/day (as sodium selenite) for 8 weeks caused large increases in cytotoxic T cells and Natural Killer (NK) cells by upregulating receptors for the growth regulatory lymphokine, interleukin-2 and consequently, the rate of cell proliferation and differentiation into cytotoxic cells [72]. One trial revealed enhancement of NK cells following selenium supplementation [73]. It follows that the “functional exhaustion” of such cells in severe COVID-19 disease may represent a reversible attenuation of selenoprotein function.

Selenium deficiency, on the other hand, seems to favour a particular balance of Th1:Th2 responses, that can be modified towards a predominant Th1 phenotype by supra-nutritional selenium supplementation [15]. This may be due to an increase in levels of free thiols following selenium supplementation and improved T-cell proliferation, which fosters a phenotypically different inflammasome [74]. This may be a crucial insight, as it has been shown that cytokine release varies significantly depending on the T-helper phenotype response in infection with respiratory-syncytial virus in children [75]. In selenium-replete individuals, Th1 predominance following polio immunisation signalled a more “robust” response to the vaccine [76].

These effects seem most striking in the elderly, who we know are overrepresented in COVID-19 mortality figures [47]. One randomised trial revealed significant increases in CD4 (+) T Cells, following prolonged supplementation with 400 μg selenium/day in elderly volunteers [77]. This indicates that the elderly, in particular, can have striking variations in their immunophenotype, which can be modulated by external factors such as selenium intake.

The effect that selenium may have on Th1:Th2 ratios needs further investigation; while a Th1 phenotype can be beneficial in developing cellular immunity, it is also associated with many of the cytokines that correlate with COVID-19 severity [78]. Whether selenium supplementation can promote a Th1 response that results in a beneficial cellular response without contributing to further inflammation, remains to be seen.

Within the innate immune system, selenium supplementation has been shown to affect macrophage responses, shifting them away from a “pro-inflammatory” reaction with regard to cytokine release [79]. In mice inoculated with Influenza A, selenium deficiency led to higher rates of macrophage infiltration of the lungs than in selenium-replete mice [80]. In one recent murine study, selenium deficiency was shown to inhibit macrophage phagocytosis directly and promote NF-κB-mediated inflammation [81].

### 4.2 Effect of selenium on inflammatory cytokine release

A cytokine storm has been identified as a pathogenic mechanism for the deterioration of critically ill patients with COVID-19 by careful examination of circulating cytokines [82]; in particular, the cytokines IL-1β and IL-6 have been recognised as leading to lung inflammation [83]. Accordingly, IL-6 levels were noted to be higher in patients with severe disease than in those with mild disease [84] and high IL-6 levels positively correlated with severe disease [83]. In previous models of pneumonia, mortality has also been shown to be associated with high IL-6 levels [85]. IL-6 is an inflammatory cytokine that plays a central role in the coordination of innate and adaptive immunity. Its inhibition has been central to the management of various manifestations of the cytokine storm [86]. Elevation of IL-6 was associated with mortality in infection with the similar coronavirus, MERS-CoV, responsible for Middle East Respiratory Syndrome (MERS) [87].

There are multiple ongoing trials regarding the use of Tocilizumab (a humanised monoclonal antibody which binds soluble and membrane-bound IL-6 receptors) in COVID-19 and some promising results, albeit in small groups, have been shown as reviewed by Zhang et al. [88]. Hydroxychloroquine, a drug that has been investigated in COVID-19, is also known to modulate the cytokine response and reduce IL-6 levels [89], though recent observational studies have not identified a clear benefit in COVID-19 [90].

Selenium has been found to downregulate the IL-6 response [43,54] and selenium deficiency has been noted to be associated with higher levels of IL-6 in the elderly [91]. *In vitro*, bronchial epithelial cells infected with Influenza A grown in selenium-deficient conditions had increased IL-6 production [92]. Induction of IL-6 in mice placed under oxidative stress was prevented by selenium supplementation [93]. Similarly, selenium-deficient mice infected by the bacterium *Listeria monocytogenes* had significantly higher circulating IL-6 levels than infected controls [94]. In a different murine trial, however, selenium adequacy led to higher levels of IL-6 in healthy mice [95]. This may indicate that an adequate level of selenium contributes to optimal levels of IL-6, both in health and disease, though further investigation is warranted.

IL-6 is a well-established surrogate for chronic inflammation in prostate cancer and inversely correlates with tumour response to chemotherapy [96]. In such cases, it has been shown that IL-6 levels can be reduced by sodium selenite supplementation [97]. In liver cirrhosis, subsequent to alcoholic liver disease, serum selenium concentration was inversely correlated with serum IL-6 [98]. One large review detailed an increase in inflammatory cytokines in the gastrointestinal system resulting from selenium deficiency [99]. In inflammatory bowel disease (IBD), selenium deficiency exacerbates colitis, as reviewed by Kudva et al. [100]. In acute myeloid and lymphoblastic leukaemias (AML and ALL), the rates of oral mucositis, following hematopoietic stem-cell transplantation (HSCT) in selenium-supplemented patients were significantly reduced compared to those of placebo controls in a randomised controlled trial [101]. Though, in this case there was no significant difference in the circulating cytokine levels, it was concluded that selenium had offset parts of the inflammatory cascade.

In Kashin-Beck disease, increased levels of IL-6, IL-1β and TNF-α were found in selenium-depleted individuals [102]. In a systematic review of 32 studies of autoimmune diseases, such as rheumatoid arthritis and vasculitis, significantly lower serum selenium concentration was identified in cases than in controls [103], though the direction of causality was unclear.

In critically ill patients with sepsis, selenium concentrations were noted to be low, and oxidative damage was higher than in replete patients [104]. Most suggestively, in a study of critically ill patients with Acute Respiratory Distress Syndrome (ARDS), selenium significantly “restored the antioxidant capacity of the lungs, moderated the inflammatory responses, and meaningfully improved the respiratory mechanics” [105]. No discernible survival benefit was appreciated, however, in that study.

The above examples appear to demonstrate that higher selenium status, or selenium supplementation, reduces the level of inflammatory cytokines; this observation requires an attempt to explain the mechanism which we have attempted to do below.

### 4.3 Selenoproteins in cytokine regulation

The activation of inflammatory cytokines, including IL-6, is coordinated by the transcription factor NF-κB, which has been shown to be specifically inhibited by selenite in cell culture studies [106]. Furthermore, IL-6 downregulates hepatic biosynthesis of a number of selenoproteins [43,54]. Hydrogen peroxide, lipid and phospholipid hydroperoxides are ROS that are reduced by the GPX family to harmless water or alcohols, thus reducing oxidative stress. Unregulated ROS production may activate NF-κB and promote excessive cytokine release [107] as well as specifically contributing to the pathogenesis of COVID-19-related acute respiratory distress syndrome (ARDS), by increasing the tendency for thrombotic micronangiopathy [108]. As NF-κB is crucial for transcription of inflammatory cytokines associated with severe COVID-19 [109], further trials are needed to elucidate whether selenium supplementation can downregulate NF-κB expression *in vivo* and indeed whether this confers a survival benefit.

Lastly, as further evidence for the importance of selenium in cytokine regulation, one selenoprotein gene *(SELENOS*), has a particular 105G/A promoter polymorphism (rs28665122), known to be strongly associated with circulating levels of inflammatory cytokines, especially IL-1β, IL-6, and TNF-α [25].

Examples in the literature have delineated a clear role for selenium in the regulation of inflammatory cytokines and implicated selenium deficiency in the pathogenesis of inflammatory states. Whether these findings reflect a hidden variable correlating with both is unestablished; however, a clear inverse correlation between selenium status and IL-6 levels has been demonstrated.

### 4.4 Viral targeting of selenoprotein mRNAs by antisense and related mechanisms

There are several possible viral mechanisms that could directly affect the host selenoproteome, thereby indirectly modifying the pool of redox-active selenium metabolites and contributing to the observed clinical benefits of selenium in certain viral infections. One is the possibility of virally-encoded glutathione peroxidases (vGPX), which would directly compete with the host for limited pools of selenocysteine in infected cells. As reviewed by Guillin et al. [33], vGPX have been demonstrated in both DNA and RNA viruses. The precedent of an HIV-1 encoded plasma GPX homologue [[110], [111], [112]] is particularly relevant for other RNA viruses like SARS-CoV-2, because current evidence suggests that the putative selenocysteine-encoding UGA codon in the viral mRNA is recoded using a SECIS element hijacked from a TXNRD1 mRNA that is captured *via* a virus/host RNA/RNA-antisense tethering interaction [41]. That microRNA-like interaction is very likely to lead additionally to knockdown of TXNRD1 protein synthesis, which is consistent with the substantial decrease in ^75^Se-labeled TXNRD1 protein levels observed in HIV-1 infected cells [113].

Computational and *in vitro* evidence support the possibility of similar antisense targeting of TXNRD mRNAs by a number of RNA viruses ([114] and references therein). In some cases, including HIV-1 and Ebola Zaire (EBOV), these interaction sites are associated with highly conserved UGA stop codons that terminate known viral open reading frames (HIV-1 nef, EBOV nucleoprotein), and so may be serving the function of SECIS capture *via* antisense tethering for the synthesis of viral selenoprotein modules [41,114,115]. In other cases (e.g. Zika virus, mumps virus and some pathogenic strains of avian influenza), there is *no* evidence of viral selenoprotein encoding, so the antisense interaction appears to serve some other function, for which the most obvious hypothesis involves the role of TXNRD in DNA synthesis [114,116]. Just as some large DNA viruses encode their own ribonucleotide reductase to increase DNA synthesis for virus production, some RNA viruses may attempt to boost RNA synthesis by inhibiting the diversion of ribonucleotides for DNA synthesis, for which thioredoxin serves as an electron donor for ribonucleotide reductase, which thus in turn requires TXNRD for sustained conversion of ribonucleotides to deoxyribonucleotides.

Based on computational analysis, like EBOV and mumps virus, SARS-CoV-2 appears to target TXNRD3 via antisense at several sites, and the quality of the interactions (22 base pairs over a range of 23 or 24 bases) is similar to that of the strongest microRNA interactions [114]. This suggests a probable knockdown of TXNRD3 at both the mRNA and protein levels, which has been validated in a recent study showing that TXNRD3 mRNA is decreased by about 37% in SARS-CoV-2 infected Vero cells [40]. Although TXNRD3 mRNA levels are highest in the testes, according to the Human Protein Atlas [117], TXNRD3 *protein* levels are as high or higher in the lung and GI tract, which are major sites of SARS-CoV-2 replication. Significantly, the ACE2 receptor used by SARS-CoV-2 is also expressed at high levels in the testes. Testicular mumps infection is classic, and EBOV infection of the testes is now understood to be a major cause of persistent infection [118]. Because of the high levels of ACE2 receptor there, SARS-CoV-2 could also target the testes. So all three of these TXNRD3-targeting viruses appear to at least have the potential to infect the tissue in which TXNRD3 is most highly expressed in human males [114].

A similar viral mechanism that is at least theoretically possible is the targeting of host selenoproteins for degradation by proteolysis, achieving a similar result as antisense knockdown, but by a different mechanism. Targeting of host proteins by viral proteases is a well-documented phenomenon but has until now never been observed *vs*. a host selenoprotein. However, the recent demonstration of a high quality protein-protein interaction between an inactive C145A mutant of the SARS-CoV-2 main cysteine protease, M^pro^, and human GPX1 [119], although rather paradoxical because of the failure to observe any interaction between wild type M^pro^ and GPX1, raises the possibility that GPX1 may be a substrate for M^pro^, and that the products of proteolytic cleavage had disassociated from the active enzyme prior to detection. This interpretation is strengthened by the identification of potential M^pro^ cleavage site sequences in GPX1 as well as in TXNRD1 and SELENOF, with the latter being essentially identical to a known M^pro^ cleavage site over a span of 8 residues [120]. Proteolytic targeting of SELENOF would complement its 76% knockdown at the mRNA level by SARS-CoV-2, suggesting that the viral agenda is significantly enhanced by interference in the function of this particular selenoprotein.

The location of the predicted M^pro^ cleavage site in TXNRD1, five residues from its C-terminal, would remove its selenocysteine-containing redox centre, making it incapable of regenerating reduced thioredoxin [120]. Along with the demonstrated knockdown of TXNRD3 at the mRNA level, this outcome is consistent with the hypothesis that SARS-CoV-2 may be actively inhibiting DNA synthesis, which would result in the increased availability of ribonucleotides for viral RNA synthesis [114,120]. Taken together, these proposed mechanisms, targeting selenoproteins at both the mRNA and protein levels, would represent an unprecedented frontal assault on selenoprotein biosynthesis by a pathogen, and suggest a potentially significant role of selenium status in the pathogenesis of COVID-19.

### 4.5 SARS-CoV-2 main protease as a target for inhibition: effectiveness of selenium-containing ebselen

The SARS-CoV-2 replicase gene encodes two overlapping polyproteins for viral replication and transcription. The polyproteins undergo extensive proteolytic processing that is largely carried out by M^pro^ to form functional polypeptides. M^pro^ plays a vital role in mediating the life cycle of SARS-CoV-2 and is an attractive target for antiviral drug design [121]. A fluorescence resonance energy transfer assay has been developed for high-throughput screening of M^pro^ inhibitors. Based on this method, 10,000 compounds, including FDA-approved drugs, clinical trial/preclinical drug candidates and natural products, have been examined for their potency of inhibiting M^pro^ activity [121]. Ebselen, an organoselenium compound designed as a GPX1 mimic, was found to have the strongest inhibitory activity [121]. Ebselen covalently binds to sulfhydryl group of the Cys145 residue in the catalytic dyad of the protease. Consistently, quantitative real-time RT-PCR showed that ebselen was also the strongest antiviral compound in SARS-CoV-2 infected Vero cells [121]. Like SARS-CoV-2 M^pro^, the papain-like protease (PL^pro^) of SARS-CoV-2 is also a cysteine protease that is pivotal for virus replication by processing the viral polyprotein into mature, functional subunits. In addition, SARS-CoV-2 uses PL^pro^ to antagonize the host's antiviral innate immune response hence it is another promising target for suppression. Ebselen was found to be a highly active inhibitor of SARS-CoV-2 PL^pro^ via covalent bind of the selenium in ebselen with the sulfhydryl group of the Cys112 residue in the catalytic triad of the protease, with an inhibition constant in the low micromolar range [122].

### 4.6 Ebselen causes thiol oxidation of critical thiol-dependent pathogen enzymes

Ebselen was found to be safe and effective in clinical trials for treatment of hearing loss and bipolar disorders [[123], [124], [125], [126]]. It possesses anti-inflammatory, anti-oxidant and cytoprotective properties by acting mainly as a peroxiredoxin mimic and to a lesser extent as a glutathione peroxidase mimic to scavenge hydrogen peroxide and peroxynitrite in mammalian cells [127]. In addition to its canonical antioxidant roles, ebselen is also viewed as a thiol-peroxidase mimic causing thiol oxidation [123,127]. Ebselen induces cellular apoptosis through rapid depletion of intracellular thiols [128,129] and is a superfast thioredoxin oxidant [130]. It also inhibits a number of thiol-dependent enzymes by targeting their critical thiol residues [[131], [132], [133]]. The inhibition can be prevented by the addition of reducing agents such as dithiothreitol. Ebselen has been found to have therapeutic potential against many infectious diseases. In most cases, ebselen acts as a covalent inhibitor of numerous thiol-dependent enzymes or functional proteins in various pathogens [[134], [135], [136], [137], [138], [139], [140], [141], [142], [143], [144], [145], [146]]. The selenol derived from ebselen is reactive to cysteine residues in proteins. The ebselen-mediated inhibition is usually dependent on the selenosulfide formed between ebselen and protein thiols [127]. Notably, ebselen has been found to be active in 193 out of 1023 drug screenings [146], revealing its promiscuous binding property and limited druggability. However, ebselen as a paradigm lead compound for inhibiting M^pro^ activity of SARS-CoV-2 and suppressing the life cycle of SARS-CoV-2 among 10,000 compounds examined [121], suggests that redox-active selenium compounds formed *in vivo* might participate in inhibiting SARS-CoV-2 replication since this class of selenium species can behave as ebselen by reacting with sulfhydryl group of protein cysteine residues (see below).

### 4.7 Redox-active selenium metabolites are involved in the anti-viral action of selenium in mice and humans

Steinbrenner et al. have predicted that effective selenium intervention in infectious diseases and cancers would have a similar dose range [42]. Cancer-preventive effects of selenium were more likely to be found at supranutritional dose levels (1–2 μg selenium/g diet) in numerous animal studies. The underlying mechanism has mainly been ascribed to the accumulation of redox-active selenium compounds at supranutritional dose levels, which largely exceed the requirement for selenoprotein biosynthesis (0.1 μg selenium/g diet) [[147], [148], [149], [150], [151]]. Anti-viral effects of selenium in animals have never been studied at such high dose levels. However, a study investigating the dose-response relationship of selenite against H1N1 influenza virus up to a maximum of 0.5 μg selenium/g diet, suggests that redox-active selenium metabolites play a role in the anti-viral action of selenium [152]. While H1N1 influenza virus resulted in a low survival percentage in selenium-deficient mice, i.e. 25%, the survival percentage was increased to 41%, 50%, 75% and 75% in mice that consumed diets long-term containing 0.2, 0.3, 0.4 and 0.5 μg selenium/g, respectively, in the form of selenite, with a Pearson r as high as 0.97 (p < 0.01), linking survival percentage to dietary selenium levels [152]. Moreover, the therapeutic effect of a short course of high-dose selenium in patients with “epidemic hemorrhagic fever” (one of several illnesses caused by hantavirus infection) also implies that redox-active selenium metabolites are involved in the anti-viral action of selenium [46]. Specifically, cases of “epidemic hemorrhagic fever” were treated exclusively by multiple oral doses of 2 mg sodium selenite/d (913 μg selenium/d) in the first 9 days of hospitalisation; compared to the untreated control, the mortality dropped from 100% to 36% in the fulminant type (p = 0.013 by Fisher's exact test) [46]. Given the aforementioned high correlation between dietary selenium (from nutritional to apparently supranutritional levels) and survival percentage following viral infection in mice [152], the dramatic reduction of hantavirus-associated fatality rate in humans after the treatment with a pharmacological dose of selenite [46], and the fact that the cure rate of COVID-19 patients continued to rise beyond the selenium intake required to optimise selenoprotein requirements [7], it is anticipated that redox-active metabolites of supranutritional selenium could also inhibit the M^pro^ of SARS-CoV-2.

### 4.8 Redox-active selenium species may have the potential to react with SARS-CoV-2 M^pro^

**Fig. 2:**
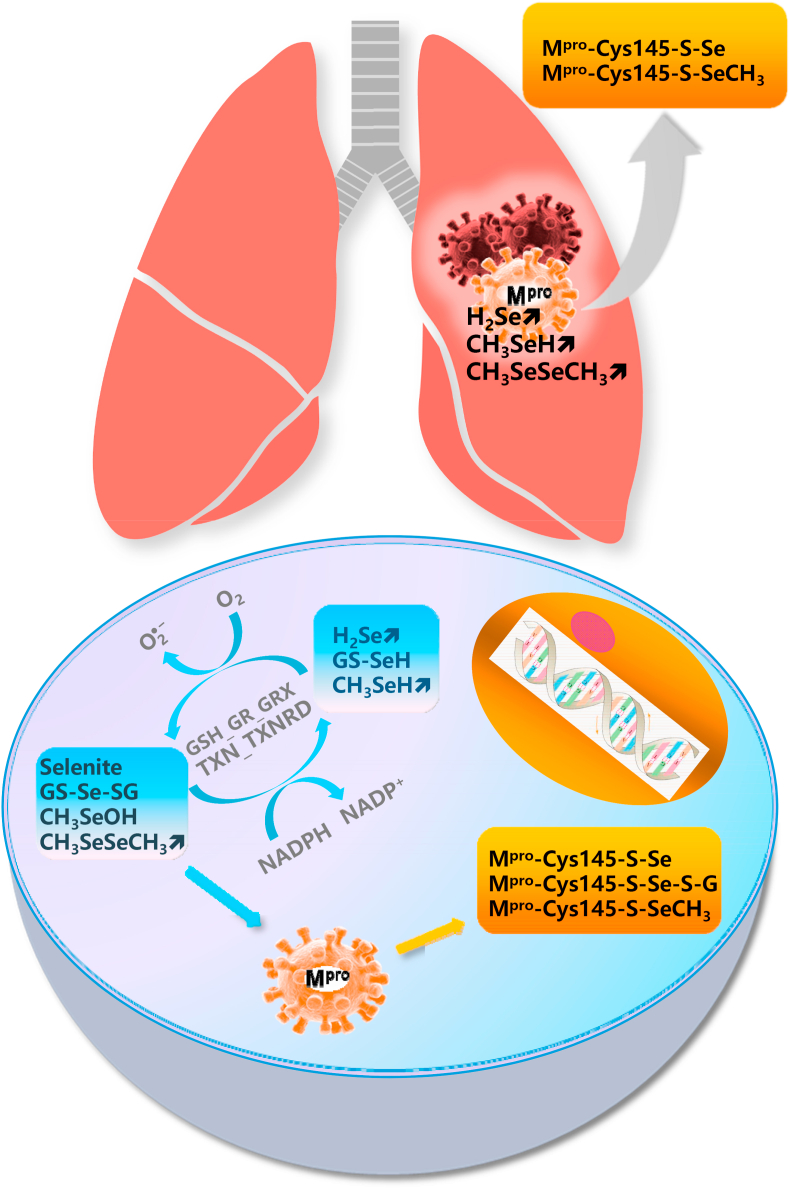
Hypothesised interaction of selenium compounds with SARS-CoV-2 consisting of cycling of redox-active selenium compounds in various cells (lower panel) and the presence of volatile redox-active selenium compounds in the lung (upper panel). GS-Se-SG, selenodiglutathione; CH_3_SeOH, methylseleninic acid; CH_3_SeSeCH_3_, dimethyldiselenide; H_2_Se, hydrogen selenide; GS-SeH, glutathione selenopersulfide; CH_3_SeH, methylselenol; GSH, glutathione; GR, GSH reductase; GRX, glutaredoxin; TXN, thioredoxin; TXNRD, TXN reductase.

Following a high intake of dietary selenium or supranutritional selenium supplementation beyond the requirement for selenoprotein biosynthesis, small molecular weight selenium compounds are accumulated [[147], [148], [149], [150], [151]]. Hydrogen selenide, methylselenol, dimethylselenide and dimethyldiselenide are volatile small molecules and thus are likely to be found in the respiratory tract [147,153,154]. Some selenium compounds such as selenite and methylseleninic acid (a precursor of methylselenol) and many intermediates of selenium metabolism including selenodiglutathione, dimethyldiselenide and elemental selenium nanoparticles have strong redox activity [[155], [156], [157]]. A common property of these selenium species is that they undergo redox cycling in the thioredoxin system, composed of thioredoxin and TXNRD, and the glutaredoxin-coupled glutathione system composed of glutathione, glutathione reductase and glutaredoxin, with the formation of ROS with the sacrifice of NADPH [[158], [159], [160]] (Fig. 2, low panel). Ganther proposed that thiol modification induced by redox-active selenium compounds might occur via modification of protein cysteine residues, leading to the formation of selenium adducts of the selenotrisulfide (S–Se–S) or selenenylsulfide (S–Se) type or disulfides [161]. Evidence supporting this biochemical deduction includes, but is not limited to: (i) Selenite and selenodiglutathione are efficient oxidants of thioredoxin [162]; (ii) Selenite inactivates intracellular caspase-3 and inhibits the activity of purified recombinant caspase-3 by modification of a critical cysteine residue present within the active site [163]; (iii) Selenite also inactivates c-Jun N-terminal kinase by a direct modification of the Cys116 residue – the inhibitory action is abolished by replacement of Cys116 by serine or the addition of reducing agents [164]; (iv) Selenite also inactivates NF-κB by modification of the essential thiols of this transcription factor [106]; (v) Methylseleninic acid induces NAD(P)H:quinone oxidoreductase-1 expression through oxidizing thiols of Keap1 to release/activate NF-E2-related factor 2. This induction was abrogated by pre-treatment of cells with dithiothreitol [165]; (vi) Locally formed methylseleninic acid, following the oxidation of methylselenol by lipid hydroperoxides bound to protein kinase C, inhibits protein kinase C activity by oxidizing the vicinal thiols present within the catalytic domain [153,[166], [167], [168]]; (vii) Global redox modification of proteins by methylseleninic acid was characterized by a display thiol-proteomics approach [169]. Given that the Cys145 residue of the M^pro^ of SARS-CoV-2 is a vital target for inhibition and the excellent performance of ebselen in this context, it is conceivable that redox-active selenium compounds could react with HS-Cys145-M^pro^ (Fig. 2, low panel), leading to reduced replication, transcription and a truncated life cycle of SARS-CoV-2.

### 4.9 The selenium metabolite, methylseleninic acid, may accumulate in SARS-CoV-2 infected cells

Gopalakrishna et al. proposed an oxidant-associated sequestration of methylselenol in cells [153]. In normal cells with lower levels of oxidants, volatile methylselenol is not oxidized to non-volatile methylseleninic acid and thus is not retained by normal cells. In promoting pre-cancer cells with higher concentrations of oxidants, volatile methylselenol is retained after conversion to non-volatile methylseleninic acid. Viral respiratory infections are known to suppress antioxidant enzymes and induce ROS generating enzymes, resulting in enhanced ROS production and increased accumulation of oxidation products which, as discussed, may play a significant role in the inflammatory response to COVID-19 [170]. Under these circumstances, it is suggested that methylselenol is converted to methylseleninic acid. The accumulated methylseleninic acid, in turn, might inactivate the M^pro^ of SARS-CoV-2 in infected cells by modifying the Cys145 residue of the M^pro^.

### 4.10 Volatile metabolites of selenium may gather in the lung

It is known that dimethyselenide compounds are expelled in the breath [171]. Jüliger et al. found that of two known dimethyselenide compounds, namely dimethylselenide and dimethyldiselenide, dimethyldiselenide was generated more rapidly and copiously than dimethylselenide in cells subjected to methylseleninic acid treatment [172]. Dimethylselenide is more than 500-times less toxic than selenite in rats [173,174], implying that it is not a typical redox-active selenium compound. However, like many diselenides, including naturally occurring selenocystine and synthetic diethyldiselenide, dipropyldiselenide and dibutyldiselenide [175] and the well-characterized diphenyldiselenide [176], dimethyldiselenide is also a redox-active selenium compound that generates ROS in the presence of glutathione [156]. The released dimethyldiselenide, in combination with methylselenol and hydrogen selenide from various cell types in many tissues, could conceivably gather in the lung, the main battlefield of viral respiratory infections, to inhibit the M^pro^ of SARS-CoV-2 (Fig. 2, upper panel). It is plausible that volatile redox-active selenium compounds formed *in vivo* following high dietary intake of selenium or high-dose selenium supplementation could constitute a defence against COVID-19 in the lung.

## 5 Concluding remarks

Most (though not all [177]) studies have demonstrated that selenium deficiency leads to increased host-susceptibility to viral infections. However, it is important to keep in mind that the effects of selenium deficiency on a virally-infected host may depend on the type of pathology induced.

With regard to effects that are likely to be primarily mediated through host selenoproteins, there is considerable evidence that a dysregulated innate immune system and cytokine release have the capacity to worsen COVID-19 [[67], [68], [69]]. In the elderly, who are particularly at risk, selenium deficiency is correlated with higher circulating inflammatory cytokines [91]. Published data support the hypothesis that selenium adequacy prevents excessive cytokine activation in infectious, inflammatory and oncological models [[82], [83], [84], [85], [86], [87], [88], [89], [90], [91]]. In some cases, supra-therapeutic selenium contributed to enhanced adaptive immunity by reinvigorating cytotoxic cells and moderating the release of inflammatory cytokines by the innate immune system [14,58]. The presence of *SELENOS* polymorphisms that directly influence cytokine production is particularly telling [24]. The promising results of selenium supplementation shown in critically-ill patients may have resulted from these complex immunomodulatory actions.

Further analysis of immunological phenomena in COVID-19, stratified by baseline selenium status, would be valuable. Whether supplementation of selenium during SARS-COV-2 infection could confer a survival benefit by dampening an overwhelming immune response is an important consideration for future clinical trials.

**Fig. 3:**
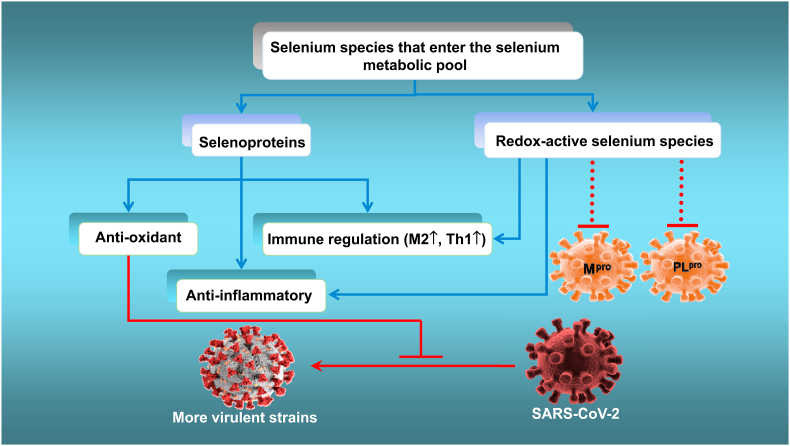
Proposed mechanism by which supranutritional levels of selenium might suppress the life cycle and mutation to virulence of SARS-COV-2 while attenuating viral-induced oxidative stress, organ damage and the cytokine storm.

Based on all the evidence described above, both selenoproteins and redox-active selenium species (that mimic selenium-containing ebselen [121]) in the selenium metabolic pool could employ their separate mechanisms to attenuate virus-triggered oxidative stress, excessive inflammatory responses and immune-system dysfunction, thus improving the outcome of SARS-CoV-2 infection, as hypothesised in Fig. 3. A possible area for future study will be the strategies that viruses use to interfere with selenium-based host-protective mechanisms.

Finally, it is well documented that there is a narrow dose range spanning the beneficial and adverse effects of selenium [3,11]. In general, the current literature discourages selenium-adequate individuals from increasing their selenium intake to a level normally associated with toxicity that would markedly increase the formation of redox-active selenium species [3,11,178]. However, for COVID-19 disease, we have identified an association between more-than-adequate selenium intake/status and higher cure rate [7]. The acute infection phase in COVID-19 is only a few weeks in typical cases, which is comparable to the time-frame over which daily doses of 1 mg selenium (as selenite) have been used in sepsis and critical care applications [105,179,180]. Based on such precedents, over a similar time-frame, a comparable supranutritional dose of selenium would be very unlikely to result in toxicity in COVID-19 patients and might be beneficial in those with moderate-to-severe symptoms. However, the potential benefit of such a strategy would need to be tested clinically, preferably in a randomised, controlled trial.

## Authors’ contributions

All authors drafted sections of the manuscript. MPR prepared Table 1 and Fig. 1. Fig. 2, Fig. 3 were prepared by JZ. The online Supplemental file was prepared by MPR and JZ. All authors revised the manuscript to produce the final version.

## Funding

There is no funding source associated with this article.

## Declaration of competing interest

None of the authors has any conflict of interests.

## References

[1] Institute of Medicine (Us) (2000). Panel on Dietary Antioxidants and Related Compounds, Dietary Reference Intakes for Vitamin C, Vitamin E, Selenium, and Carotenoids.

[2] Department of Health (1991). Dietary reference values of the committee on medical aspects of food policy (COMA). Dietary Reference Values for Food, Energy and Nutrients for the United Kingdom.

[3] Rayman M.P. (2012). Selenium and human health. Lancet.

[4] Johnson C.C., Fordyce F.M., Rayman M.P. (2010). Symposium on 'Geographical and geological influences on nutrition': factors controlling the distribution of selenium in the environment and their impact on health and nutrition. Proc. Nutr. Soc..

[5] Blazina T., Sun Y., Voegelin A., Lenz M., Berg M., Winkel L.H. (2014). Terrestrial selenium distribution in China is potentially linked to monsoonal climate. Nat. Commun..

[6] Winther K.H., Rayman M.P., Bonnema S.J., Hegedus L. (2020). Selenium in thyroid disorders - essential knowledge for clinicians. Nat. Rev. Endocrinol..

[7] Zhang J., Taylor E.W., Bennett K., Saad R., Rayman M.P. (2020). Association between regional selenium status and reported outcome of COVID-19 cases in China. Am. J. Clin. Nutr..

[8] Xia Y., Hill K.E., Li P., Xu J., Zhou D., Motley A.K., Wang L., Byrne D.W., Burk R.F. (2010). Optimization of selenoprotein P and other plasma selenium biomarkers for the assessment of the selenium nutritional requirement: a placebo-controlled, double-blind study of selenomethionine supplementation in selenium-deficient Chinese subjects. Am. J. Clin. Nutr..

[9] Yang G.Q., Xia Y.M. (1995). Studies on human dietary requirements and safe range of dietary intakes of selenium in China and their application in the prevention of related endemic diseases. Biomed. Environ. Sci..

[10] Fairweather-Tait S.J., Bao Y., Broadley M.R., Collings R., Ford D., Hesketh J.E., Hurst R. (2011). Selenium in human health and disease. Antioxidants Redox Signal..

[11] Rayman M.P. (2020). Selenium intake, status, and health: a complex relationship. Hormones (Basel).

[12] Labunskyy V.M., Hatfield D.L., Gladyshev V.N. (2014). Selenoproteins: molecular pathways and physiological roles. Physiol. Rev..

[13] Kohrle J. (2005). Selenium and the control of thyroid hormone metabolism. Thyroid.

[14] Qian F., Misra S., Prabhu K.S. (2019). Selenium and selenoproteins in prostanoid metabolism and immunity. Crit. Rev. Biochem. Mol. Biol..

[15] Huang Z., Rose A.H., Hoffmann P.R. (2012). The role of selenium in inflammation and immunity: from molecular mechanisms to therapeutic opportunities. Antioxidants Redox Signal..

[16] Beck M.A., Handy J., Levander O.A. (2004). Host nutritional status: the neglected virulence factor. Trends Microbiol..

[17] Xiong Y.M., Mo X.Y., Zou X.Z., Song R.X., Sun W.Y., Lu W., Chen Q., Yu Y.X., Zang W.J. (2010). Association study between polymorphisms in selenoprotein genes and susceptibility to Kashin-Beck disease. Osteoarthritis Cartilage.

[18] Peng X., Lin Y., Li J., Liu M., Wang J., Li X., Liu J., Jia X., Jing Z., Huang Z. (2016). Evaluation of glutathione peroxidase 4 role in preeclampsia. Sci. Rep..

[19] Conrad M., Ingold I. (2018). Selenium and iron, two elemental rivals in the ferroptotic death process. Oncotarget.

[20] Muri J., Heer S., Matsushita M., Pohlmeier L., Tortola L., Fuhrer T., Conrad M., Zamboni N., Kisielow J., Kopf M. (2018). The thioredoxin-1 system is essential for fueling DNA synthesis during T-cell metabolic reprogramming and proliferation. Nat. Commun..

[21] Yoshioka J. (2015). Thioredoxin reductase 2 (Txnrd2) regulates mitochondrial integrity in the progression of age-related heart failure. J Am Heart Assoc.

[22] Rayman M.P., Hatfield D.L., Berry M.J., Gladyshev V.N. (2011). Selenium and adverse conditions of human pregnancy. Selenium: its Molecular Biology and Role in Human Health.

[23] McKenzie R.C., Arthur J.R., Beckett G.J. (2002). Selenium and the regulation of cell signaling, growth, and survival: molecular and mechanistic aspects. Antioxidants Redox Signal..

[24] Kirkby K.A., Adin A.C. (2006). Products of heme oxygenase and their potential therapeutic applications. Am. J. Physiol. Ren. Physiol..

[25] Curran J.E., Jowett J.B., Elliott K.S., Gao Y., Gluschenko K., Wang J., Abel Azim D.M., Cai G., Mahaney M.C., Comuzzie A.G. (2005). Genetic variation in selenoprotein S influences inflammatory response. Nat. Genet..

[26] Santos L.R., Duraes C., Mendes A., Prazeres H., Alvelos M.I., Moreira C.S., Canedo P., Esteves C., Neves C., Carvalho D. (2014). A polymorphism in the promoter region of the selenoprotein S gene (SEPS1) contributes to Hashimoto's thyroiditis susceptibility. J. Clin. Endocrinol. Metab..

[27] Moses E.K., Johnson M.P., Tømmerdal L., Forsmo S., Curran J.E., Abraham L.J., Charlesworth J.C., Brennecke S.P., Blangero J., Austgulen R. (2008). Genetic association of preeclampsia to the inflammatory response gene SEPS1. Am. J. Obstet. Gynecol..

[28] Alanne M., Kristiansson K., Auro K., Silander K., Kuulasmaa K., Peltonen L., Salomaa V., Perola M. (2007). Variation in the selenoprotein S gene locus is associated with coronary heart disease and ischemic stroke in two independent Finnish cohorts. Hum. Genet..

[29] Roy M., Kiremidjian-Schumacher L. (1998). Selenium and immune function. Z. Ernahrungswiss..

[30] Pitts M.W., Hoffmann P.R. (2018). Endoplasmic reticulum-resident selenoproteins as regulators of calcium signaling and homeostasis. Cell Calcium.

[31] Rayman M.P. (2019). Multiple nutritional factors and thyroid disease, with particular reference to autoimmune thyroid disease. Proc. Nutr. Soc..

[32] Matsushita M., Freigang S., Schneider C., Conrad M., Bornkamm G.W., Kopf M. (2015). T cell lipid peroxidation induces ferroptosis and prevents immunity to infection. J. Exp. Med..

[33] Guillin O.M., Vindry C., Ohlmann T., Chavatte L. (2019). Selenium, selenoproteins and viral infection. Nutrients.

[34] Sousa V.C., Carmo R.F., Vasconcelos L.R., Aroucha D.C., Pereira L.M., Moura P., Cavalcanti M.S. (2016). Association of catalase and glutathione peroxidase 1 polymorphisms with chronic hepatitis C outcome. Ann. Hum. Genet..

[35] Hiscott J., Kwon H., Genin P. (2001). Hostile takeovers: viral appropriation of the NF-kappaB pathway. J. Clin. Invest..

[36] Burk R.F., Hill K.E. (2015). Regulation of selenium metabolism and transport. Annu. Rev. Nutr..

[37] Blankenberg S., Rupprecht H.J., Bickel C., Torzewski M., Hafner G., Tiret L., Smieja M., Cambien F., Meyer J., Lackner K.J. (2003). Glutathione peroxidase 1 activity and cardiovascular events in patients with coronary artery disease. N. Engl. J. Med..

[38] Tang N.P., Wang L.S., Yang L., Gu H.J., Sun Q.M., Cong R.H., Zhou B., Zhu H.J., Wang B. (2008). Genetic variant in glutathione peroxidase 1 gene is associated with an increased risk of coronary artery disease in a Chinese population. Clin. Chim. Acta.

[39] Crosley L.K., Bashir S., Nicol F., Arthur J.R., Hesketh J.E., Sneddon A.A. (2013). The single-nucleotide polymorphism (GPX4c718t) in the glutathione peroxidase 4 gene influences endothelial cell function: interaction with selenium and fatty acids. Mol. Nutr. Food Res..

[40] Wang Y., Huang J., Sun Y., He J., Li W., Liu Z., Taylor E.W., Rayman M.P., Wan X., Zhang J. (2020). SARS-CoV-2 suppresses mRNA expression of selenoproteins associated with ferroptosis, ER stress and DNA synthesis. BioRxiv.

[41] Taylor E.W., Ruzicka J.A., Premadasa L., Zhao L. (2016). Cellular selenoprotein mRNA tethering via antisense interactions with Ebola and HIV-1 mRNAs may impact host selenium biochemistry. Curr. Top. Med. Chem..

[42] Steinbrenner H., Al-Quraishy S., Dkhil M.A., Wunderlich F., Sies H. (2015). Dietary selenium in adjuvant therapy of viral and bacterial infections. Adv Nutr.

[43] Hoffmann P.R., Berry M.J. (2008). The influence of selenium on immune responses. Mol. Nutr. Food Res..

[44] Baum M.K., Shor-Posner G., Lai S., Zhang G., Lai H., Fletcher M.A., Sauberlich H., Page J.B. (1997). High risk of HIV-related mortality is associated with selenium deficiency. J. Acquir. Immune Defic. Syndr. Hum. Retrovirol..

[45] Yu S.Y., Zhu Y.J., Li W.G. (1997). Protective role of selenium against hepatitis B virus and primary liver cancer in Qidong. Biol. Trace Elem. Res..

[46] Hou J.C. (1997). Inhibitory effect of selenite and other antioxidants on complement-mediated tissue injury in patients with epidemic hemorrhagic fever. Biol. Trace Elem. Res..

[47] Williamson E.J., Walker A.J., Bhaskaran K., Bacon S., Bates C., Morton C.E., Curtis H.J., Mehrkar A., Evans D., Inglesby P. (2020). Factors associated with COVID-19-related death using OpenSAFELY. Nature.

[48] Takahashi T., Ellingson M.K., Wong P., Israelow B., Lucas C., Klein J., Silva J., Mao T., Oh J.E., Tokuyama M. (2020). Sex differences in immune responses that underlie COVID-19 disease outcomes. Nature.

[49] Peng Y., Meng K., He M., Zhu R., Guan H., Ke Z., Leng L., Wang X., Liu B., Hu C. (2020). Clinical Characteristics and Prognosis of 244 Cardiovascular Patients Suffering from Coronavirus Disease in Wuhan, China.

[50] Santos M.C., Oliveira A.L., Viegas-Crespo A.M., Vicente L., Barreiros A., Monteiro P., Pinheiro T., Bugalho De Almeida A. (2004). Systemic markers of the redox balance in chronic obstructive pulmonary disease. Biomarkers.

[51] Robberecht H., De Bruyne T., Davioud-Charvet E., Mackrill J., Hermans N. (2019). Selenium status in elderly people: longevity and age-related diseases. Curr. Pharmaceut. Des..

[52] Nichol C., Herdman J., Sattar N., O’Dwyer P.J., O’Reilly D.St.J., Littlejohn D., Fell G. (1998). Changes in the concentrations of plasma selenium and selenoproteins after minor elective surgery: further evidence for a negative acute phase response?. Clin. Chem..

[53] Hesse-Bahr K., Dreher I., Kohrle J. (2000). The influence of the cytokines Il-1beta and INFgamma on the expression of selenoproteins in the human hepatocarcinoma cell line HepG2. Biofactors.

[54] Martitz J., Becker N.P., Renko K., Stoedter M., Hybsier S., Schomburg L. (2015). Gene-specific regulation of hepatic selenoprotein expression by interleukin-6. Metall.

[55] Huang Y., Wang Q., Gao J., Lin Z., Banuelos G.S., Yuan L., Yin X. (2013). Daily dietary selenium intake in a high selenium area of Enshi, China. Nutrients.

[56] Dinh Q.T., Cui Z., Huang J., Tran T.A.T., Wang D., Yang W., Zhou F., Wang M., Yu D., Liang D. (2018). Selenium distribution in the Chinese environment and its relationship with human health: a review. Environ. Int..

[57] Li S., Banuelos G.S., Wu L., Shi W. (2014). The changing selenium nutritional status of Chinese residents. Nutrients.

[58] Hurst R., Armah C.N., Dainty J.R., Hart D.J., Teucher B., Goldson A.J., Broadley M.R., Motley A.K., Fairweather-Tait S.J. (2010). Establishing optimal selenium status: results of a randomized, double-blind, placebo-controlled trial. Am. J. Clin. Nutr..

[59] Seale LA T.D., Berry M.J., Pitts M.W. (2020). A Role for Selenium-dependent GPX1 in SARS-CoV-2 Virulence.

[60] Johns Hopkins Coronavirus Resource Center Mortality analyses. https://coronavirus.jhu.edu/data/mortality.

[61] Stoffaneller R., Morse N.L. (2015). A review of dietary selenium intake and selenium status in Europe and the Middle East. Nutrients.

[62] Moghaddam A., Heller R.A., Sun Q., Seelig J., Cherkezov A., Seibert L., Hackler J., Seemann P., Diegmann J., Pilz M. (2020). Selenium deficiency is associated with mortality risk from COVID-19. Nutrients.

[63] Chen M., Zhou W., Xu W. (2020). Thyroid Function Analysis in 50 Patients with COVID-19: A Retrospective Study.

[64] Ye Q., Wang B., Mao J. (2020). Cytokine storm in COVID-19 and treatment. J. Infect..

[65] Zheng M., Gao Y., Wang G., Song G., Liu S., Sun D., Xu Y., Tian Z. (2020). Functional exhaustion of antiviral lymphocytes in COVID-19 patients. Cell. Mol. Immunol..

[66] Zhang W., Zhao Y., Zhang F., Wang Q., Li T., Liu Z., Wang J., Qin Y., Zhang X., Yan X. (2020). The use of anti-inflammatory drugs in the treatment of people with severe coronavirus disease 2019 (COVID-19): the Perspectives of clinical immunologists from China. Clin. Immunol..

[67] Zhang Y., Xiao M., Zhang S., Xia P., Cao W., Jiang W., Chen H., Ding X., Zhao H., Zhang H. (2020). Coagulopathy and antiphospholipid antibodies in patients with covid-19. N. Engl. J. Med..

[68] Toscano G., Palmerini F., Ravaglia S., Ruiz L., Invernizzi P., Cuzzoni M.G., Franciotta D., Baldanti F., Daturi R., Postorino P. (2020). Guillain–barré syndrome associated with SARS-CoV-2. N. Engl. J. Med..

[69] Zulfiqar A.-A., Lorenzo-Villalba N., Hassler P., Andrès E. (2020). Immune thrombocytopenic Purpura in a patient with covid-19. N. Engl. J. Med..

[70] Galván Casas C., Català A., Carretero Hernández G., Rodríguez-Jiménez P., Fernández Nieto D., Rodríguez-Villa Lario A., Navarro Fernández I., Ruiz-Villaverde R., Falkenhain D., Llamas Velasco M. (2020). Classification of the cutaneous manifestations of COVID-19: a rapid prospective nationwide consensus study in Spain with 375 cases. Br. J. Dermatol..

[71] Huang H., Jiao X., Xu Y., Han Q., Jiao W., Liu Y., Li S., Teng X. (2019). Dietary selenium supplementation alleviates immune toxicity in the hearts of chickens with lead-added drinking water. Avian Pathol..

[72] Kiremidjian-Schumacher L., Roy M., Wishe H.I., Cohen M.W., Stotzky G. (1994). Supplementation with selenium and human immune cell functions. II. Effect on cytotoxic lymphocytes and natural killer cells. Biol. Trace Elem. Res..

[73] Ravaglia G., Forti P., Maioli F., Bastagli L., Facchini A., Mariani E., Savarino L., Sassi S., Cucinotta D., Lenaz G. (2000). Effect of micronutrient status on natural killer cell immune function in healthy free-living subjects aged >/=90 y. Am. J. Clin. Nutr..

[74] Hoffmann F.W., Hashimoto A.C., Shafer L.A., Dow S., Berry M.J., Hoffmann P.R. (2010). Dietary selenium modulates activation and differentiation of CD4+ T cells in mice through a mechanism involving cellular free thiols. J. Nutr..

[75] Yuan X.-h., Li Y.-m., Shen Y.-y., Yang J., Jin Y. (2020). Clinical and Th1/Th2 immune response features of hospitalized children with human rhinovirus infection. J. Med. Virol..

[76] Broome C.S., McArdle F., Kyle J.A., Andrews F., Lowe N.M., Hart C.A., Arthur J.R., Jackson M.J. (2004). An increase in selenium intake improves immune function and poliovirus handling in adults with marginal selenium status. Am. J. Clin. Nutr..

[77] Wood S.M., Beckham C., Yosioka A., Darban H., Watson R.R. (2000). beta-Carotene and selenium supplementation enhances immune response in aged humans. Integr. Med..

[78] Romagnani S. (2000). T-cell subsets (Th1 versus Th2). Ann. Allergy Asthma Immunol..

[79] Nelson S.M., Lei X., Prabhu K.S. (2011). Selenium levels affect the IL-4-induced expression of alternative activation markers in murine macrophages. J. Nutr..

[80] Beck M.A., Nelson H.K., Shi Q., Van Dael P., Schiffrin E.J., Blum S., Barclay D., Levander O.A. (2001). Selenium deficiency increases the pathology of an influenza virus infection. Faseb. J..

[81] Xu J., Gong Y., Sun Y., Cai J., Liu Q., Bao J., Yang J., Zhang Z. (2020). Impact of selenium deficiency on inflammation, oxidative stress, and phagocytosis in mouse macrophages. Biol. Trace Elem. Res..

[82] Huang C., Wang Y., Li X., Ren L., Zhao J., Hu Y., Zhang L., Fan G., Xu J., Gu X. (2020). Clinical features of patients infected with 2019 novel coronavirus in Wuhan, China. Lancet.

[83] Conti P., Ronconi G., Caraffa A., Gallenga C.E., Ross R., Frydas I., Kritas S.K. (2020). Induction of pro-inflammatory cytokines (IL-1 and IL-6) and lung inflammation by Coronavirus-19 (COVI-19 or SARS-CoV-2): anti-inflammatory strategies. J. Biol. Regul. Homeost. Agents.

[84] Wan S., Yi Q., Fan S., Lv J., Zhang X., Guo L., Lang C., Xiao Q., Xiao K., Yi Z. (2020). Characteristics of lymphocyte subsets and cytokines in peripheral blood of 123 hospitalized patients with 2019 novel coronavirus pneumonia (NCP). medRxiv.

[85] Bacci M.R., Leme R.C., Zing N.P., Murad N., Adami F., Hinnig P.F., Feder D., Chagas A.C., Fonseca F.L. (2015). IL-6 and TNF-alpha serum levels are associated with early death in community-acquired pneumonia patients. Braz. J. Med. Biol. Res..

[86] Kishimoto T., Narazaki M. (2018). The two-faced cytokine IL-6 in host defense and diseases. Int. J. Mol. Sci..

[87] Fehr A.R., Channappanavar R., Perlman S. (2017). Middle East respiratory syndrome: emergence of a pathogenic human coronavirus. Annu. Rev. Med..

[88] Zhang C., Wu Z., Li J.W., Zhao H., Wang G.Q. (2020). The cytokine release syndrome (CRS) of severe COVID-19 and Interleukin-6 receptor (IL-6R) antagonist Tocilizumab may be the key to reduce the mortality. Int. J. Antimicrob. Agents.

[89] Sahraei Z., Shabani M., Shokouhi S., Saffaei A. (2020). Aminoquinolines against coronavirus disease 2019 (COVID-19): chloroquine or hydroxychloroquine. Int. J. Antimicrob. Agents.

[90] Geleris J., Sun Y., Platt J., Zucker J., Baldwin M., Hripcsak G., Labella A., Manson D.K., Kubin C., Barr R.G. (2020). Observational study of hydroxychloroquine in hospitalized patients with covid-19. N. Engl. J. Med..

[91] Tseng C.K., Ho C.T., Hsu H.S., Lin C.H., Li C.I., Li T.C., Liu C.S., Lin C.C., Lin W.Y. (2013). Selenium is inversely associated with interleukin-6 in the elderly. J. Nutr. Health Aging.

[92] Jaspers I., Zhang W., Brighton L.E., Carson J.L., Styblo M., Beck M.A. (2007). Selenium deficiency alters epithelial cell morphology and responses to influenza. Free Radic. Biol. Med..

[93] Viezeliene D., Beekhof P., Gremmer E., Rodovicius H., Sadauskiene I., Jansen E., Ivanov L. (2013). Selective induction of IL-6 by aluminum-induced oxidative stress can be prevented by selenium. J. Trace Elem. Med. Biol..

[94] Wang C., Wang H., Luo J., Hu Y., Wei L., Duan M., He H. (2009). Selenium deficiency impairs host innate immune response and induces susceptibility to Listeria monocytogenes infection. BMC Immunol..

[95] Tsuji P.A., Carlson B.A., Anderson C.B., Seifried H.E., Hatfield D.L., Howard M.T. (2015). Dietary selenium levels affect selenoprotein expression and support the interferon-γ and IL-6 immune response pathways in mice. Nutrients.

[96] Nguyen D.P., Li J., Tewari A.K. (2014). Inflammation and prostate cancer: the role of interleukin 6 (IL-6). BJU Int..

[97] Gazi M.H., Gong A., Donkena K.V., Young C.Y. (2007). Sodium selenite inhibits interleukin-6-mediated androgen receptor activation in prostate cancer cells via upregulation of c-Jun. Clin. Chim. Acta.

[98] Prystupa A., Kiciński P., Luchowska-Kocot D., Błażewicz A., Niedziałek J., Mizerski G., Jojczuk M., Ochal A., Sak J.J., Załuska W. (2017). Association between serum selenium concentrations and levels of proinflammatory and profibrotic cytokines-interleukin-6 and growth differentiation factor-15, in patients with alcoholic liver cirrhosis. Int. J. Environ. Res. Publ. Health.

[99] Nettleford S.K., Prabhu K.S. (2018). Selenium and selenoproteins in gut inflammation-A review. Antioxidants.

[100] Kudva A.K., Shay A.E., Prabhu K.S. (2015). Selenium and inflammatory bowel disease. Am. J. Physiol. Gastrointest. Liver Physiol..

[101] Daeian N., Radfar M., Jahangard-Rafsanjani Z., Hadjibabaie M., Ghavamzadeh A. (2014). Selenium supplementation in patients undergoing hematopoietic stem cell transplantation: effects on pro-inflammatory cytokines levels. Daru.

[102] Zhou X., Wang Z., Chen J., Wang W., Song D., Li S., Yang H., Xue S., Chen C. (2014). Increased levels of IL-6, IL-1beta, and TNF-alpha in Kashin-Beck disease and rats induced by T-2 toxin and selenium deficiency. Rheumatol. Int..

[103] Sahebari M., Rezaieyazdi Z., Khodashahi M. (2019). Selenium and autoimmune diseases: a review article. Curr. Rheumatol. Rev..

[104] Mertens K., Lowes D.A., Webster N.R., Talib J., Hall L., Davies M.J., Beattie J.H., Galley H.F. (2015). Low zinc and selenium concentrations in sepsis are associated with oxidative damage and inflammation. Br. J. Anaesth..

[105] Mahmoodpoor A., Hamishehkar H., Shadvar K., Ostadi Z., Sanaie S., Saghaleini S.H., Nader N.D. (2019). The effect of intravenous selenium on oxidative stress in critically ill patients with acute respiratory distress syndrome. Immunol. Invest..

[106] Kim I.Y., Stadtman T.C. (1997). Inhibition of NF-kappaB DNA binding and nitric oxide induction in human T cells and lung adenocarcinoma cells by selenite treatment. Proc. Natl. Acad. Sci. U. S. A..

[107] Tattoli I., Carneiro L.A., Jéhanno M., Magalhaes J.G., Shu Y., Philpott D.J., Arnoult D., Girardin S.E. (2008). NLRX1 is a mitochondrial NOD-like receptor that amplifies NF-kappaB and JNK pathways by inducing reactive oxygen species production. EMBO Rep..

[108] Qiao J., Arthur J.F., Gardiner E.E., Andrews R.K., Zeng L., Xu K. (2018). Regulation of platelet activation and thrombus formation by reactive oxygen species. Redox Biol.

[109] Ghosh S., Hayden M.S. (2011). NF-κB in immunobiology. Cell Res..

[110] Taylor E.W., Bhat A., Nadimpalli R.G., Zhang W., Kececioglu J. (1997). HIV-1 encodes a sequence overlapping env gp41 with highly significant similarity to selenium-dependent glutathione peroxidases. J. Acquir. Immune Defic. Syndr. Hum. Retrovirol..

[111] Zhao L., Cox A.G., Ruzicka J.A., Bhat A.A., Zhang W., Taylor E.W. (2000). Molecular modeling and in vitro activity of an HIV-1-encoded glutathione peroxidase. Proc. Natl. Acad. Sci. U. S. A..

[112] Cohen I., Boya P., Zhao L., Métivier D., Andreau K., Perfettini J.L., Weaver J.G., Badley A., Taylor E.W., Kroemer G. (2004). Anti-apoptotic activity of the glutathione peroxidase homologue encoded by HIV-1. Apoptosis.

[113] Gladyshev V.N., Stadtman T.C., Hatfield D.L., Jeang K.T. (1999). Levels of major selenoproteins in T cells decrease during HIV infection and low molecular mass selenium compounds increase. Proc. Natl. Acad. Sci. U. S. A..

[114] Taylor E.W. (2020). RNA viruses vs. DNA synthesis: a general viral strategy that may contribute to the protective antiviral effects of selenium. Preprints.

[115] Taylor E.W., Ruzicka J.A., Premadasa L. (2015). Translational readthrough of the Ebola nucleoprotein 3’-UGA codon via antisense tethering of thioredoxin reductase 3 mRNA. International Congress on Targeting Ebola.

[116] Taylor E.W., Ruzicka J.A. (2016). Zika-mediated antisense inhibition of selenoprotein synthesis may contribute to neurologic disorders and microcephaly by mimicking SePP1 knockout and the genetic disease PCCA. Zika Open Preprint Server, Bull. World Health Organ..

[117] Uhlén M., Fagerberg L., Hallström B.M., Lindskog C., Oksvold P., Mardinoglu A., Sivertsson Å., Kampf C., Sjöstedt E., Asplund A. (2015). Proteomics. Tissue-based map of the human proteome. Science.

[118] Schindell B.G., Webb A.L., Kindrachuk J. (2018). Persistence and sexual transmission of filoviruses. Viruses.

[119] Gordon D.E., Jang G.M., Bouhaddou M., Xu J., Obernier K., White K.M., O’Meara M.J., Rezelj V.V., Guo J.Z., Swaney D.L. (2020). A SARS-CoV-2 protein interaction map reveals targets for drug repurposing. Nature.

[120] Taylor E.W., Radding, W. (2020). Understanding selenium and glutathione as antiviral factors in COVID-19: does the viral M^pro^ protease target host selenoproteins and glutathione synthesis?. Front Nutr.

[121] Jin Z., Du X., Xu Y., Deng Y., Liu M., Zhao Y., Zhang B., Li X., Zhang L., Peng C. (2020). Structure of M(pro) from SARS-CoV-2 and discovery of its inhibitors. Nature.

[122] Węglarz-Tomczak E., Tomczak J.M., Talma M., Brul S. (2020). Ebselen as a Highly Active Inhibitor of PLproCoV2 bioRxiv 2020.05.17.100768.

[123] Sies H., Parnham M.J. (2013). The early research and development of ebselen. Biochem. Pharmacol..

[124] Kil J., Lobarinas E., Spankovich C., Griffiths S.K., Antonelli P.J., Lynch E.D., Le Prell C.G. (2017). Safety and efficacy of ebselen for the prevention of noise-induced hearing loss: a randomised, double-blind, placebo-controlled, phase 2 trial. Lancet.

[125] Singh N., Sharpley A.L., Emir U.E., Masaki C., Herzallah M.M., Gluck M.A., Sharp T., Harmer C.J., Vasudevan S.R., Cowen P.J. (2016). Effect of the putative lithium mimetic ebselen on brain myo-inositol, sleep, and emotional processing in humans. Neuropsychopharmacology.

[126] Nosengo N. (2016). Can you teach old drugs new tricks?. Nature.

[127] Ren X., Zou L., Lu J., Holmgren A. (2018). Selenocysteine in mammalian thioredoxin reductase and application of ebselen as a therapeutic. Free Radic. Biol. Med..

[128] Yang C.F., Shen H.M., Ong C.N. (2000). Ebselen induces apoptosis in HepG(2) cells through rapid depletion of intracellular thiols. Arch. Biochem. Biophys..

[129] Yang C.F., Shen H.M., Ong C.N. (2000). Intracellular thiol depletion causes mitochondrial permeability transition in ebselen-induced apoptosis. Arch. Biochem. Biophys..

[130] Zhao R., Masayasu H., Holmgren A. (2002). Ebselen: a substrate for human thioredoxin reductase strongly stimulating its hydroperoxide reductase activity and a superfast thioredoxin oxidant. Proc. Natl. Acad. Sci. U. S. A..

[131] Schewe C., Schewe T., Wendel A. (1994). Strong inhibition of mammalian lipoxygenases by the antiinflammatory seleno-organic compound ebselen in the absence of glutathione. Biochem. Pharmacol..

[132] Walther M., Holzhutter H.G., Kuban R.J., Wiesner R., Rathmann J., Kuhn H. (1999). The inhibition of mammalian 15-lipoxygenases by the anti-inflammatory drug ebselen: dual-type mechanism involving covalent linkage and alteration of the iron ligand sphere. Mol. Pharmacol..

[133] Hanavan P.D., Borges C.R., Katchman B.A., Faigel D.O., Ho T.H., Ma C.T., Sergienko E.A., Meurice N., Petit J.L., Lake D.F. (2015). Ebselen inhibits QSOX1 enzymatic activity and suppresses invasion of pancreatic and renal cancer cell lines. Oncotarget.

[134] Lieberman O.J., Orr M.W., Wang Y., Lee V.T. (2014). High-throughput screening using the differential radial capillary action of ligand assay identifies ebselen as an inhibitor of diguanylate cyclases. ACS Chem. Biol..

[135] Mukherjee S., Weiner W.S., Schroeder C.E., Simpson D.S., Hanson A.M., Sweeney N.L., Marvin R.K., Ndjomou J., Kolli R., Isailovic D. (2014). Ebselen inhibits hepatitis C virus NS3 helicase binding to nucleic acid and prevents viral replication. ACS Chem. Biol..

[136] Favrot L., Grzegorzewicz A.E., Lajiness D.H., Marvin R.K., Boucau J., Isailovic D., Jackson M., Ronning D.R. (2013). Mechanism of inhibition of Mycobacterium tuberculosis antigen 85 by ebselen. Nat. Commun..

[137] Chiou J., Wan S., Chan K.F., So P.K., He D., Chan E.W., Chan T.H., Wong K.Y., Tao J., Chen S. (2015). Ebselen as a potent covalent inhibitor of New Delhi metallo-beta-lactamase (NDM-1). Chem Commun (Camb).

[138] Bender K.O., Garland M., Ferreyra J.A., Hryckowian A.J., Child M.A., Puri A.W., Solow-Cordero D.E., Higginbottom S.K., Segal E., Banaei N. (2015). A small-molecule antivirulence agent for treating Clostridium difficile infection. Sci. Transl. Med..

[139] Cryan L.M., Habeshian K.A., Caldwell T.P., Morris M.T., Ackroyd P.C., Christensen K.A., Rogers M.S. (2013). Identification of small molecules that inhibit the interaction of TEM8 with anthrax protective antigen using a FRET assay. J. Biomol. Screen.

[140] Thenin-Houssier S., de Vera I.M., Pedro-Rosa L., Brady A., Richard A., Konnick B., Opp S., Buffone C., Fuhrmann J., Kota S. (2016). Ebselen, a small-molecule capsid inhibitor of HIV-1 replication. Antimicrob. Agents Chemother..

[141] Eltahan R., Guo F., Zhang H., Xiang L., Zhu G. (2018). Discovery of ebselen as an inhibitor of Cryptosporidium parvum glucose-6-phosphate isomerase (CpGPI) by high-throughput screening of existing drugs. Int J Parasitol Drugs Drug Resist.

[142] Gordhan H.M., Patrick S.L., Swasy M.I., Hackler A.L., Anayee M., Golden J.E., Morris J.C., Whitehead D.C. (2017). Evaluation of substituted ebselen derivatives as potential trypanocidal agents. Bioorg. Med. Chem. Lett.

[143] Lu J., Vlamis-Gardikas A., Kandasamy K., Zhao R., Gustafsson T.N., Engstrand L., Hoffner S., Engman L., Holmgren A. (2013). Inhibition of bacterial thioredoxin reductase: an antibiotic mechanism targeting bacteria lacking glutathione. Faseb. J..

[144] Gustafsson T.N., Osman H., Werngren J., Hoffner S., Engman L., Holmgren A. (2016). Ebselen and analogs as inhibitors of Bacillus anthracis thioredoxin reductase and bactericidal antibacterials targeting Bacillus species, Staphylococcus aureus and Mycobacterium tuberculosis. Biochim. Biophys. Acta.

[145] Sarwono A.E.Y., Mitsuhashi S., Kabir M.H.B., Shigetomi K., Okada T., Ohsaka F., Otsuguro S., Maenaka K., Igarashi M., Kato K. (2019). Repurposing existing drugs: identification of irreversible IMPDH inhibitors by high-throughput screening. J. Enzym. Inhib. Med. Chem..

[146] Zhang D.W., Yan H.L., Xu X.S., Xu L., Yin Z.H., Chang S., Luo H. (2020). The selenium-containing drug ebselen potently disrupts LEDGF/p75-HIV-1 integrase interaction by targeting LEDGF/p75. J. Enzym. Inhib. Med. Chem..

[147] Ip C. (1998). Lessons from basic research in selenium and cancer prevention. J. Nutr..

[148] Ip C. (1981). Prophylaxis of mammary neoplasia by selenium supplementation in the initiation and promotion phases of chemical carcinogenesis. Canc. Res..

[149] Combs G.F., Gray W.P. (1998). Chemopreventive agents: selenium. Pharmacol. Ther..

[150] Combs G.F., Clark L.C., Turnbull B.W. (2001). An analysis of cancer prevention by selenium. Biofactors.

[151] Jiang C., Wang Z., Ganther H., Lü J. (2002). Distinct effects of methylseleninic acid versus selenite on apoptosis, cell cycle, and protein kinase pathways in DU145 human prostate cancer cells. Mol. Canc. Therapeut..

[152] Yu L., Sun L., Nan Y., Zhu L.Y. (2011). Protection from H1N1 influenza virus infections in mice by supplementation with selenium: a comparison with selenium-deficient mice. Biol. Trace Elem. Res..

[153] Gopalakrishna R., Gundimeda U., Zhou S., Zung K., Forell K., Holmgren A. (2016). Imbalance in protein thiol redox regulation and cancer-preventive efficacy of selenium. React Oxyg Species (Apex).

[154] Ohta Y., Kobayashi Y., Konishi S., Hirano S. (2009). Speciation analysis of selenium metabolites in urine and breath by HPLC- and GC-inductively coupled plasma-MS after administration of selenomethionine and methylselenocysteine to rats. Chem. Res. Toxicol..

[155] Misra S., Boylan M., Selvam A., Spallholz J.E., Bjornstedt M. (2015). Redox-active selenium compounds--from toxicity and cell death to cancer treatment. Nutrients.

[156] Spallholz J.E., Shriver B.J., Reid T.W. (2001). Dimethyldiselenide and methylseleninic acid generate superoxide in an in vitro chemiluminescence assay in the presence of glutathione: implications for the anticarcinogenic activity of L-selenomethionine and L-Se-methylselenocysteine. Nutr. Canc..

[157] Zhao G., Wu X., Chen P., Zhang L., Yang C.S., Zhang J. (2018). Selenium nanoparticles are more efficient than sodium selenite in producing reactive oxygen species and hyper-accumulation of selenium nanoparticles in cancer cells generates potent therapeutic effects. Free Radic. Biol. Med..

[158] Fernandes A.P., Wallenberg M., Gandin V., Misra S., Tisato F., Marzano C., Rigobello M.P., Kumar S., Bjornstedt M. (2012). Methylselenol formed by spontaneous methylation of selenide is a superior selenium substrate to the thioredoxin and glutaredoxin systems. PloS One.

[159] Wallenberg M., Olm E., Hebert C., Bjornstedt M., Fernandes A.P. (2010). Selenium compounds are substrates for glutaredoxins: a novel pathway for selenium metabolism and a potential mechanism for selenium-mediated cytotoxicity. Biochem. J..

[160] Wu X., Zhao G., He Y., Wang W., Yang C.S., Zhang J. (2019). Pharmacological mechanisms of the anticancer action of sodium selenite against peritoneal cancer in mice. Pharmacol. Res..

[161] Ganther H.E. (1999). Selenium metabolism, selenoproteins and mechanisms of cancer prevention: complexities with thioredoxin reductase. Carcinogenesis.

[162] Ren X., Bjornstedt M., Shen B., Ericson M.L., Holmgren A. (1993). Mutagenesis of structural half-cystine residues in human thioredoxin and effects on the regulation of activity by selenodiglutathione. Biochemistry.

[163] Park H.S., Huh S.H., Kim Y., Shim J., Lee S.H., Park I.S., Jung Y.K., Kim I.Y., Choi E.J. (2000). Selenite negatively regulates caspase-3 through a redox mechanism. J. Biol. Chem..

[164] Park H.S., Park E., Kim M.S., Ahn K., Kim I.Y., Choi E.J. (2000). Selenite inhibits the c-Jun N-terminal kinase/stress-activated protein kinase (JNK/SAPK) through a thiol redox mechanism. J. Biol. Chem..

[165] Park J.M., Kim D.H., Na H.K., Surh Y.J. (2018). Methylseleninic acid induces NAD(P)H:quinone oxidoreductase-1 expression through activation of NF-E2-related factor 2 in Chang liver cells. Oncotarget.

[166] Gundimeda U., Gopalakrishna R. (2002). Antioxidant regulation of protein kinase C in cancer prevention. J. Nutr..

[167] Gopalakrishna R., Gundimeda U., Zhou S., Bui H., Holmgren A. (2018). Redox regulation of protein kinase C by selenometabolites and selenoprotein thioredoxin reductase limits cancer prevention by selenium. Free Radic. Biol. Med..

[168] Gundimeda U., Schiffman J.E., Chhabra D., Wong J., Wu A., Gopalakrishna R. (2008). Locally generated methylseleninic acid induces specific inactivation of protein kinase C isoenzymes: relevance to selenium-induced apoptosis in prostate cancer cells. J. Biol. Chem..

[169] Park E.M., Choi K.S., Park S.Y., Kong E.S., Zu K.E., Wu Y., Zhang H., Ip C., Park Y.M. (2005). A display thiol-proteomics approach to characterize global redox modification of proteins by selenium: implications for the anticancer action of selenium. CANCER GENOMICS PROTEOMICS.

[170] Khomich O.A., Kochetkov S.N., Bartosch B., Ivanov A.V. (2018). Redox biology of respiratory viral infections. Viruses.

[171] Rayman M.P., Infante H.G., Sargent M. (2008). Food-chain selenium and human health: spotlight on speciation. Br. J. Nutr..

[172] Juliger S., Goenaga-Infante H., Lister T.A., Fitzgibbon J., Joel S.P. (2007). Chemosensitization of B-cell lymphomas by methylseleninic acid involves nuclear factor-kappaB inhibition and the rapid generation of other selenium species. Canc. Res..

[173] Wilber C.G. (1980). Toxicology of selenium: a review. Clin. Toxicol..

[174] McConnell K.P., Portman O.W. (1952). Toxicity of dimethyl selenide in the rat and mouse. Proc Soc Exp Biol Med.

[175] Alvarez-Perez M., Ali W., Marc M.A., Handzlik J., Dominguez-Alvarez E. (2018). Selenides and diselenides: a review of their anticancer and chemopreventive activity. Molecules.

[176] Barbosa N.V., Nogueira C.W., Nogara P.A., de Bem A.F., Aschner M., Rocha J.B.T. (2017). Organoselenium compounds as mimics of selenoproteins and thiol modifier agents. Metall.

[177] LiM W., Beck A. (2007). Selenium deficiency induced an altered immune response and increased survival following influenza A/Puerto Rico/8/34 infection. Exp. Biol. Med..

[178] Rayman M.P., Winther K.H., Pastor-Barriuso R., Cold F., Thvilum M., Stranges S., Guallar E., Cold S. (2018). Effect of long-term selenium supplementation on mortality: results from a multiple-dose, randomised controlled trial. Free Radic. Biol. Med..

[179] Angstwurm M.W., Engelmann L., Zimmermann T., Lehmann C., Spes C.H., Abel P., Strauss R., Meier-Hellmann A., Insel R., Radke J. (2007). Selenium in Intensive Care (SIC): results of a prospective randomized, placebo-controlled, multiple-center study in patients with severe systemic inflammatory response syndrome, sepsis, and septic shock. Crit. Care Med..

[180] Manzanares W., Langlois P.L., Hardy G. (2013). Selenium pharmaconutrition in sepsis: to give or not to give? Is this still the question?. Nutrition.

