# Supplementary material for: Selenium and selenoproteins in viral infection with potential relevance to COVID-19
Source: Redox Biol. 2020 Sep 10;37:101715. doi: 10.1016/j.redox.2020.101715 (PMC7481318; doi:10.1016/j.redox.2020.101715)
Supplement: Multimedia component 1 [file mmc1.docx]

**Supplemental Material**

**Selenium and selenoproteins in viral infection with potential relevance to COVID-19**

Zhang, Saad, Taylor, Rayman

**Contents**

**Supplemental Table1:** Selenium intake in various countries taken from publications
since 2000 (except for Venezuela where data are pre-2000 but are the only data available)

**Supplemental Table 2**: Hair selenium concentration and corresponding selenium intake

**References for Supplemental Table 2**

**Supplemental Figure 1:** Linear regression of selenium intake against hair selenium concentration (using data from supplemental Table 2)

**Supplemental Table 3:** Interpolated values of hair selenium concentration as a linear function of selenium intake or vice-versa

**Supplemental Table 1: Selenium intake in various countries taken from publications
since 2000 except for Venezuela where data are pre-2000 but are the only data available** (from Winther, Rayman, Bonnema, Hegedus. Selenium in thyroid disorders - essential knowledge for clinicians*.* Nat Rev Endocrinol, 2020. 16: 165-176, with permission).

| Country | Selenium Intake (µg/d)  Minimum; Maximum  or single value | Sources |
| --- | --- | --- |
| China | 14; 550 | Huang Y, et al. *Nutrients* **8,** 700-710. doi: 10.3390/nu5030700 (2013); Xia Y, et al. *Am J Clin Nutr* **92**, 525-531. doi: 10.3945/ajcn.2010.29642 (2010). |
| Venezuela* | 752 | Rayman MP. *Br J Nutr* **100,** 254-268. doi: 10.1017/S0007114508939830 (2008). |
| Greenland | 235 | Hansen JC et al. *Sci Total Environ* **331,** 207-214 (2004). |
| Canada | 101; 190 | Hu XF, et al *Environ Int* **102,** 200-206. doi: 10.1016/j.envint.2017.03.002 (2017); Wang Y et al, *Nutrients* **4,** E24. doi: 10.3390/nu8010024 (2016). |
| Japan | 83;178 | Yoneyama S et al, *Eur J Clin Nutr* **62,** 1187-1193 (2008); Miyazaki Y et al, *J Nutr Sci Vitaminol (Tokyo)* **50,** 309-319 (2004). |
| USA | 89; 125 | [USDA 2001-2, https://www.ars.usda.gov/ARSUserFiles/80400530/pdf/0102/usualintaketables2001-02.pdf](https://www.ars.usda.gov/ARSUserFiles/80400530/pdf/0102/usualintaketables2001-02.pdf) |
| Argentina | 96 | Secchi DG et al, *Nutr Hosp* **1,** 1214-1221. doi: 10.3305/nh.2015.32.3.9277 (2015). |
| Saudi Arabia | 42; 93 | Al-Othman AM et al, *Environmen Geochem Health* **34,** 417-31. doi: 10.1007/s10653-011-9448-5 (2012); Alissa et al, *J Trace Elem Med Biol* **20,** 105-114 (2006). |
| Australia | 63; 116 | FSA Australia New Zealand 2003 |
| Spain | 21; 107 | Gonzales S et al, *Ann Nutr Metab* **50,** 126-131 (2006); Rivas A et al, *Nutr Hosp* **27,** 1886-93. doi: 10.3305/nh.2012.27.6.6039 (2012). |
| Finland | 56; 80 | Flynn A et al, *Food Nutr Res* **12,** 53. doi: 10.3402/fnr.v53i0.2038 (2009). |
| Italy | 57; 66 | Filippini T et al, *J Trace Elem Med Biol* **50,** 508-517. doi: 10.1016/j.jtemb.**2018**.03.001 (2018); Stranges S et al, *BMC Public Health* **21,** 564. doi: 10.1186/1471-2458-10-564 (2010). |
| Poland | 25; 62 | Flynn A et al, *Food Nutr Res* **12,** 53. doi: 10.3402/fnr.v53i0.2038 (2009); Skibniewska et al, *Przegl Lek* **64,** Suppl 4:15-8 (2007). |
| Belgium | 60 | Waegeneers et al, *Food Addit Contam Part A Chem Anal Control Expo Risk Assess* **30,** 278-85. doi: 10.1080/19440049.2012.746474 (2013). |
| UK | 46 | UK NDNS 2014-2016; <https://www.gov.uk/government/statistics/ndns-results-from-years-7-and-8-combined> |
| Denmark | 34; 43 | Flynn A et al, *Food Nutr Res* **12,** 53. doi: 10.3402/fnr.v53i0.2038 (2009). |
| France | 52 | Emanuelle B et al. *Environ Int* **40,** 155-161. doi: 10.1016/j.envint.2011.07.007 (2012). |
| Netherlands | 38; 51 | Flynn A et al, *Food Nutr Res* **12,** 53. doi: 10.3402/fnr.v53i0.2038 (2009). |
| Ireland | 50 | Rayman MP. *Br J Nutr* **100,** 254-268. doi: 10.1017/S0007114508939830 (2008). |
| Slovenia | 48 | Pograjc L et al, *Biol Trace Elem Res* **145,** 291-299. doi: 10.1007/s12011-011-9204-9 (2012). |
| Jordan | 59 | Arafa MA et al, *Asian Pac J Cancer Prev* **12,** 1931-1936 (2012). |
| New Zealand | 45; 58 | Thomson CD et al, *Am J Clin Nutr* **87,** 379-384 (2008); Brough L et al, *Nutrients* **9,** E254. doi: 10.3390/nu9030254 (2017). |
| Malawi | 24; 40 | Chilimba ADC et al. *Sci Rep* **1,** 72. doi: 10.1038/srep00072 (2011). |
| Iran | 35 | Nazeminezhad R et al, *Eur J Clin Nutr* **68,** 109-113. doi: 10.1038/ejcn.2013.205 (2013). |

**Supplemental Table 2.** Hair selenium concentration and corresponding

selenium intake (relevant references below)

| **City and Province** | **Weighted hair Se (mg/Kg)** | **Se intake (µg/day)** |
| --- | --- | --- |
| Guangzhou of Guangdong | 0.50 (1,2) | 98.5 (1) |
| Suzhou of Jiangsu | 0.36 (1,2) | 43.9 (1) |
| Wuxi of Jiangsu | 0.34 (1,2) | 34.7 (1) |
| Harbin of Heilongjiang | 0.26 (1-3) | 16.0 (1) |
| Suihua of Heilongjiang | 0.26 (1,2) | 16.0 (1) |
| Qiqihar of Heilongjiang | 0.26 (1,2) | 16.0 (1) |
| Beijing - Municipality | 0.57 (1,2) | 70.3 (1) |
| Xi'an of Shaanxi | 0.29 (2) | 26.0 (1) |
| Huaibei of Anhui | 0.39 (1) | 31.9 (1) |
| Nanchong of Sichuan | 0.41 (1) | 54.6 (1) |
| Xichang of Sichuan | 0.26 (1) | 11.0 (1) |

**References for Supplemental Table2**

1. Dinh QT, Cui Z, Huang J, Tran TAT, Wang D, Yang W, Zhou F, Wang M, Yu D, Liang D. Selenium distribution in the Chinese environment and its relationship with human health: A review. Environ Int. 2018; 112:294-309.
2. Li S, Banuelos GS, Wu L, Shi W. The changing selenium nutritional status of Chinese residents. Nutrients. 2014; 6:1103-14.
3. Li S, Wang T, Ye C, Li Q, Guo Z, Wu H, et al. An approach to assessment of Keshan disease elimination at the township level. Int Health. 2016; 8:398-404.

Pearson's r (95% CI) = 0.91 (0.68, 0.98)

R^2^ = 0.82

**Supplemental Figure 1.** Linear regression of selenium intake

against hair selenium concentration (using data from supplemental Table 2)

Given that GPX1 activity is maximised at an intake of 55 µg/d and plasma SELENOP concentration is maximised at an intake of 105 µg/d [Hurst et al. 2010 Am J Clin Nutr, 2010. 91(4): 923-31], from the graph of Se intake *vs* hair Se concentration (Supplemental Figure 1),

Se_intake_ = 232.98 Se_hair_ – 44.521

from which corresponding values of Se intake and hair concentration can be calculated as shown in Supplemental Table 3.

**Supplemental Table 3.** Interpolated values of hair selenium
concentration as a linear function of selenium intake or vice-versa

| **Hair Se (mg/kg)** | **Se intake (µg/day)** | **Intake corresponds to** |
| --- | --- | --- |
| 0.43 | 55 | Maximal platelet GPX1 activity |
| 0.64 | 105 | Maximal SELENOP concentration |
| 1.00 | 188 | Maximal cure rate in investigated cities  (intake at top of regression line in Figure 1) |
